# Targeting Nrf2/KEAP1 signaling pathway using bioactive compounds to combat mastitis

**DOI:** 10.3389/fimmu.2025.1425901

**Published:** 2025-02-07

**Authors:** Muhammad Zahoor Khan, Liangliang Li, Yandong Zhan, Huang Binjiang, Xiaotong Liu, Xiyan Kou, Adnan Khan, Abdul Qadeer, Qudrat Ullah, Khalid J. Alzahrani, Tongtong Wang, Changfa Wang, Muhammad Zahoor

**Affiliations:** ^1^ Liaocheng Research Institute of Donkey High-Efficiency Breeding and Ecological Feeding, Liaocheng University, Liaocheng, China; ^2^ Genome Analysis Laboratory of the Ministry of Agriculture, Agricultural Genomics Institute at Shenzhen, Chinese Academy of Agricultural Sciences, Shenzhen, China; ^3^ Department of Cell Biology, School of Life Sciences, Central South University, Changsha, China; ^4^ Department of Theriogenology, Faculty of Veterinary and Animal Sciences, Cholistan University of Veterinary and Animal Sciences, Bahawalpur, Punjab, Pakistan; ^5^ Department of Clinical Laboratories Sciences, College of Applied Medical Sciences, Taif University, Taif, Saudi Arabia; ^6^ Department of Molecular Medicine, Institute of Basic Medical Sciences, University of Oslo, Oslo, Norway

**Keywords:** mastitis, inflammation, immunity, oxidative stress, antioxidants, bioactive compounds, Nrf2/Keap1 signaling pathway

## Abstract

Mastitis is a common inflammation of mammary glands that has a significantly impact on dairy production and animal health, causing considerable economic burdens worldwide. Elevated reactive oxygen species (ROS) followed by oxidative stress, apoptosis, inflammatory changes and suppressed immunity are considered the key biomarkers observed during mastitis. The Nrf2/KEAP1 signaling pathway plays a critical role in regulating antioxidant responses and cellular defense mechanisms. When activated by bioactive compound treatment, Nrf2 translocates to the nucleus and induces the expression of its target genes to exert antioxidant responses. This reduces pathogen-induced oxidative stress and inflammation by inhibiting NF-kB signaling in the mammary glands, one of the prominent pro-inflammatory signaling pathway. Here, we summarize recent studies to highlight the therapeutic potential of Nrf2/KEAP1 pathway in the prevention and treatment of mastitis. Collectively this review article aims to explore the potential of bioactive compounds in mitigating mastitis by targeting the Nrf2/KEAP1 signaling pathway.

## 1 Introduction

Mastitis, an inflammation of the mammary glands, is characterized by increased inflammation, reactive oxygen species (ROS), and reduced immune effectiveness in the mammary gland tissues (1). This condition poses a significant challenge to the global dairy industry, leading to considerable financial burdens due to decreased milk yield, the need for therapies, reproductive issues, and the necessity for animal culling, as indicated by various studies (2–6). Globally, mastitis incurs substantial economic costs, estimated to be between US$19.7 billion and US$32 billion annually. In the United States alone, the annual economic loss due to mastitis is estimated at around US$2 billion (7, 8). In Canada, the dairy industry faces an annual financial loss of Can$400 million (equivalent to US$318 million), while in China, the estimated annual fiscal losses due to mastitis range between 15 (2.1 billion USD) and 45 (6.3 billion USD) billion Chinese Yuan (CNY) (9).

The multifaceted nature of mastitis as a disease is widely recognized in the scientific community (10). Mastitis is typically classified into clinical and sub-clinical forms. Clinical mastitis is characterized by pronounced pathological (redness, pain and fever) and physical changes (swollen and hot) in mammary gland tissues, while sub-clinical mastitis, particularly when caused by *Staphylococcus aureus*, often presents more subtly, with no obvious symptoms except for elevated milk somatic cell counts and a decrease in milk yield (11–17). The primary bacterial pathogens associated with mastitis include *Escherichia coli, Streptococcus uberis, S. dysgalactiae*, and *S. aureus* (18). The susceptibility of animals to mastitis is influenced by various factors such as the anatomical positioning of the udder, lactation stages, age, and conditions during the periparturient period (18–20).

**Figure 1:**
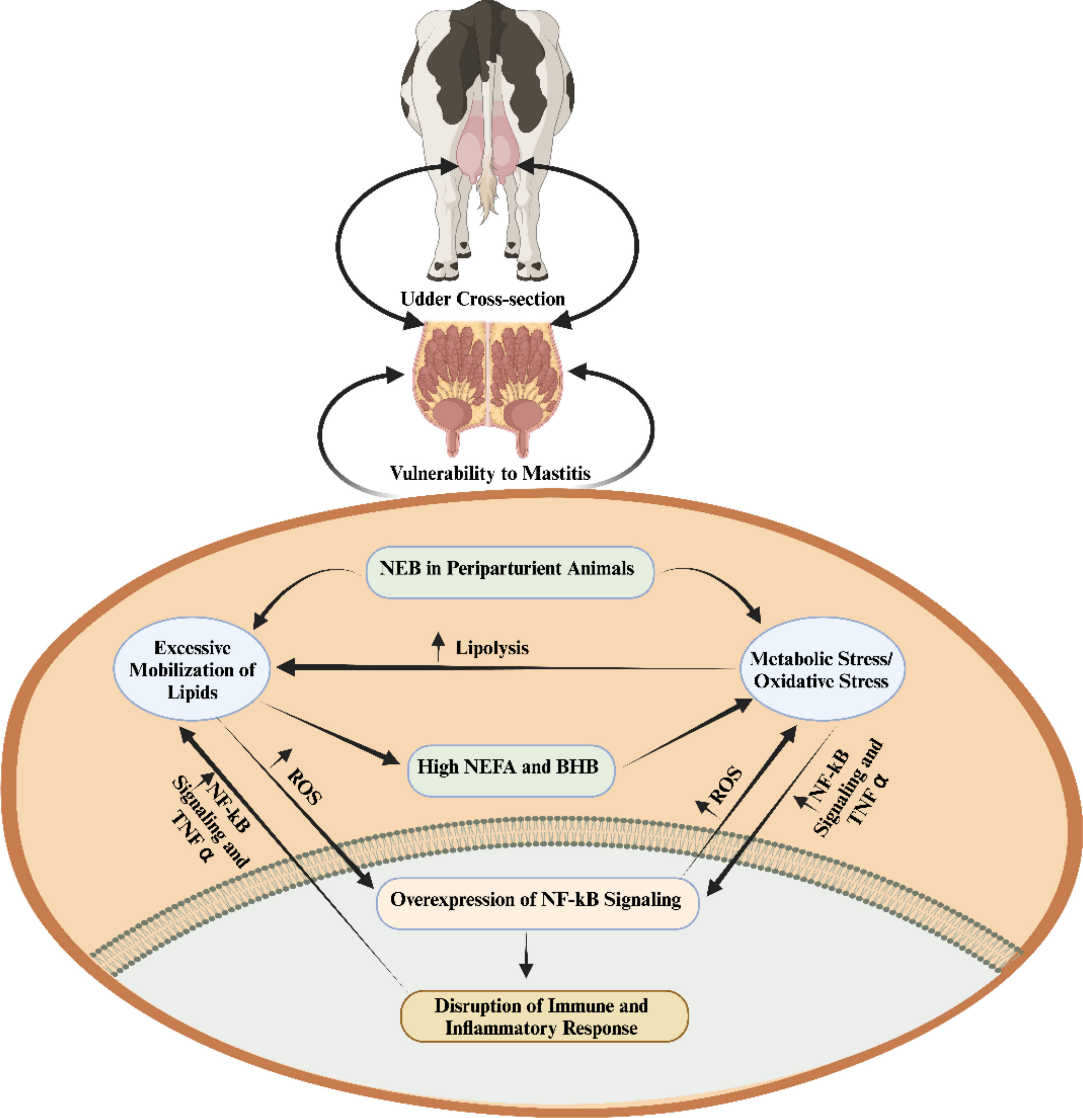
The interplay among negative energy balance induced oxidative stress, immunity and inflammation during periparturient period (1). When there is a negative energy balance, the process of lipolysis is enhanced by metabolic/oxidative stress. The excessive lipid mobilization elevates inflammation and disrupts the immunity via regulation of nuclear factor kappa-light-chain-enhancer of activated B cells (NF-kB) signaling. non-esterified fatty acids (NEFA); β-Hydroxybutyrate (BHB); tumor necrosis factor α (TNF-α); negative energy balance (NEB); reactive oxygen species (ROS).

The periparturient period is particularly critical, as animals experience a negative energy balance, leading to suppressed immunity, enhanced inflammatory responses, and an overproduction of ROS (21). This imbalance necessitates increased oxygen consumption for cellular respiration, thereby inducing oxidative stress (22). Factors such as a high body condition score (BCS), elevated levels of non-esterified fatty acids (NEFA), and β-hydroxybutyric acid (BHB) have been identified as significant contributors to the augmentation of ROS production during periparturient period (21–24). The elevated levels of oxidative stress activate the nuclear factor kappa B (NF-κB) signaling pathways, which in turn promote inflammatory changes in the mammary glands (25, 26). Additionally, the relationship between negative energy balance-induced oxidative stress, suppressed immunity, and heightened inflammatory changes is clearly depicted in **Figure 1**. Oxidative stress is a pivotal factor associated with compromised immunity and the intensification of inflammatory responses, thereby facilitate the pathogenesis of mastitis (1, 21, 22, 27). In response, several recent investigations have underscored the efficacy of antioxidant supplementation in mitigating oxidative stress and, consequently, alleviating mastitis (28–32).

Given the complex genetic mechanisms of mastitis, the erythroid-2 related factor 2/Kelch-like ECH-associated protein 1 (Nrf2/KEAP1) signaling pathway has received significant attention due to its crucial role in regulating antioxidant responses and reducing oxidative distress (33, 34). Notably, several bioactive compounds such as Metformin and Resveratrol have been demonstrated to significantly upregulate Nrf2 levels and activate antioxidant response elements, thereby attenuating oxidative stress and ameliorating mastitis induced by lipopolysaccharide (LPS) (35, 36). In light of the critical function of Nrf2/KEAP1 signaling pathway in the context of mastitis, the present study endeavors to elucidate the research trajectory concerning key pharmacological agents and antioxidants targeting this pathway as a preventative strategy against mastitis in animals.

## 2 Methodology

This review article was synthesized based on an extensive examination of literature primarily published from 2018 to 2024. Additionally, seven articles published between 2015 and 2017, and one study from 2009, were also considered for discussion in current review article. The search for relevant literature was conducted through distinguished academic databases such as X-MOL, Web of Science, Google Scholar, and PubMed. Keywords utilized in this search included ‘Nrf2/KEAP1 signaling pathway,’ ‘inflammation,’ ‘apoptosis,’ ‘antioxidant,’ ‘oxidative stress,’ ‘mastitis,’ and ‘bioactive compounds,’ with a focus on their antioxidant and anti-inflammatory properties. Inclusion criteria were restricted to articles published in journals indexed in the Science Citation Index (SCI) and in the English language. Exclusions were made for book chapters, articles published in non-SCI indexed journals, and those written in languages other than English. This methodological approach ensured a focused and comprehensive review of the relevant scientific literature.

## 3 Role of oxidative stress in the pathogenesis of mastitis

During bacterial infections, there is a marked increase in the production of ROS, which play a crucial role in pathogen clearance while also contributing significantly to the initiation and amplification of inflammatory signaling pathways (37, 38). Mastitis, often caused by bacterial pathogens such as *S. aureus or E. coli*, elicits a robust immune response. This response is primarily characterized by the recruitment and activation of innate immune cells, especially neutrophils and macrophages, at the site of infection (39, 40). These immune cells utilize ROS generation as a critical mechanism to combat invading pathogens (41–43). Immune cells like neutrophils, upon encountering pathogens, undergo a process known as the oxidative or respiratory burst. This rapid release of ROS, including hydrogen peroxide (H_2_O_2_) and superoxide radicals, acts as a powerful antimicrobial strategy aimed at destroying the invading microorganisms. However, while ROS are essential in pathogen clearance, excessive or prolonged production can result in tissue damage. In mastitis, the inflammatory process leads to the activation of endothelial cells in the mammary gland, which in turn increases vascular permeability. This heightened permeability facilitates the infiltration of immune cells to the infection site, but it also contributes to increased ROS production from both endothelial cells and the infiltrating immune cells.

**Figure 2:**
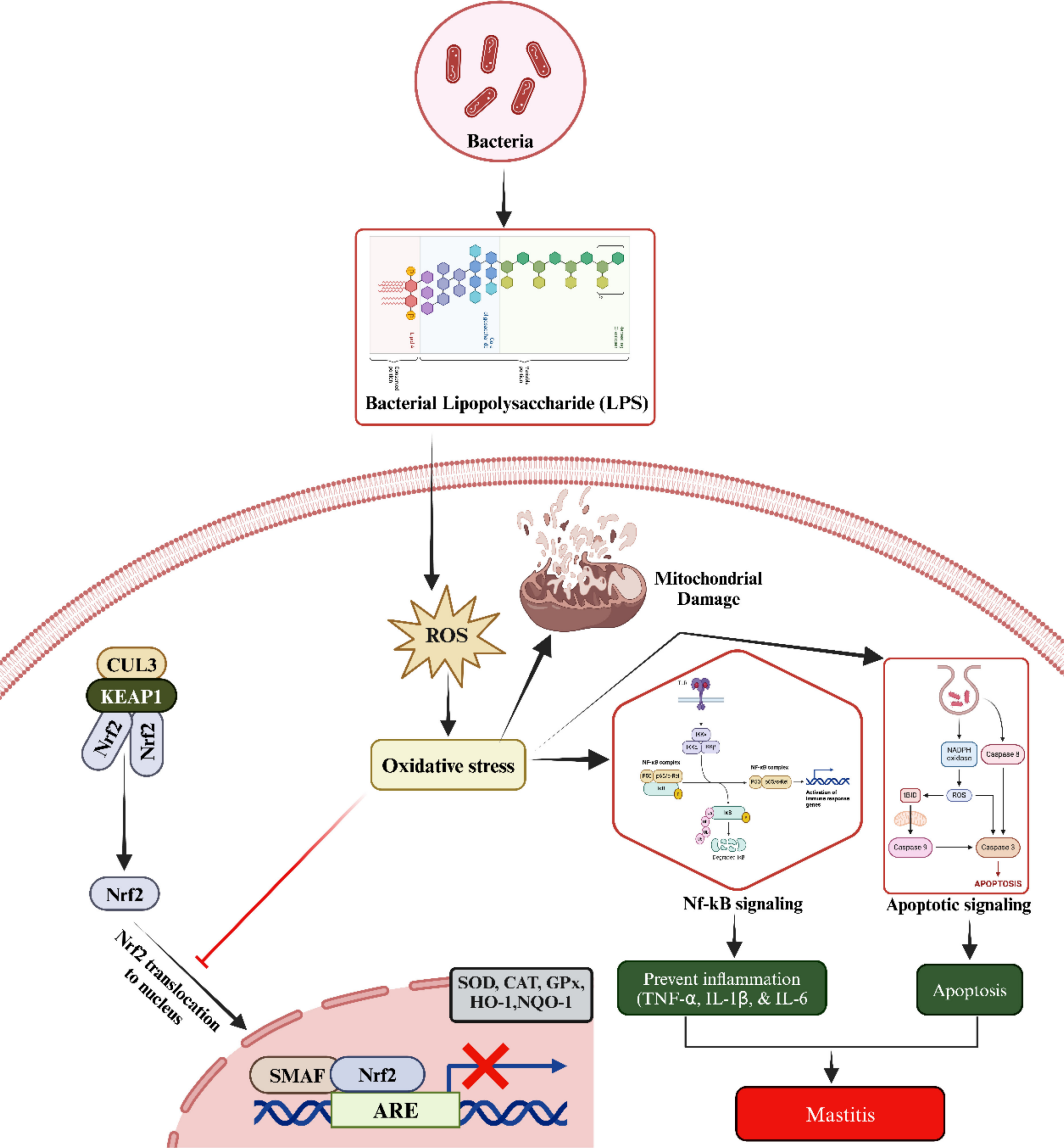
The underlined molecular mechanism during mastitis development. The LPS increased the ROS production. Elevated ROS disrupts the oxidant and antioxidant balance via blocking the entry of Nrf2 to nucleus. Additionally, elevated oxidative stress triggers the activation of NF-κB and apoptotic signaling pathways, leading to inflammatory changes, injury to mammary gland cells, and subsequent development of mastitis. Kelch-like ECH-associated protein 1 (KEAP1), tumor necrosis factor α (TNF-α), interleukin-1 β (IL-1β), and interleukin-6 (IL-6), nuclear factor erythroid 2-related factor 2 (Nrf2), cullin 3 (CUL3), heme oxygenase 1 (HO-1), antioxidant response elements (AREs), superoxide dismutase (SOD), catalase (CAT), glutathione peroxidase (GPX), and NAD(P)H quinone oxidoreductase 1 (NQO1).

Inflammation and infection also stimulate the release of various cytokines and chemokines, which are signaling molecules that regulate the immune response. Some of these molecules further activate immune cells, resulting in additional ROS production. Notably, cytokines such as tumor necrosis factor α (TNF-α), interleukin-1 β (IL-1β), and interleukin-6 (IL-6)—all of which are commonly elevated during mastitis—can enhance ROS production through immune cell activation. The combined effects of pathogen presence and immune system activation can lead to tissue damage and cellular stress within the mammary gland. Stressed and damaged cells, as a result of altered metabolic and physiological states, can produce ROS as a byproduct. Under normal physiological conditions, the body’s antioxidant defenses maintain a balance to prevent excessive ROS production and subsequent tissue damage. However, during mastitis, the increased ROS levels can overwhelm these natural defenses, leading to oxidative stress (44). In brief, when LPS enters the body, it activates Toll-like receptors (TLRs) on immune cells such as mast cells, macrophages, and epithelial cells, leading to the production of ROS. These ROS contribute to oxidative stress, which drives tissue damage and inflammation in mastitis. Elevated ROS damages cellular components, including lipids, proteins, and DNA, while also activating IKK (IκB kinase), which leads to the degradation of IκB proteins. This allows NF-κB to move into the nucleus, where it promotes the expression of pro-inflammatory cytokines like TNF-α, IL-6, and IL-1β, as well as chemokines that recruit immune cells—key events in the inflammatory phase of mastitis. Additionally, excessive ROS, often resulting from mitochondrial dysfunction and chronic inflammation, can prevent the degradation of Keap1. This inhibits NRF2 from dissociating from Keap1, impairing its ability to activate antioxidant defense mechanisms (**Figure 2**).

Several recent studies have demonstrated that LPS, not only triggers innate immune responses but also induces oxidative damage and apoptosis (45–49). The imbalance between the antioxidant capabilities of the mammary gland and the excessive ROS production—driven by the high metabolic activity of the gland—contributes significantly to the development of mastitis. This imbalance is a major factor leading to decreased milk yield and quality (50, 51). Beyond bacterial infections, other factors such as negative energy balance, heat stress, and environmental toxins can also induce oxidative stress in the mammary gland, leading to further cellular damage (28, 52–56). Given these insights, reducing oxidative stress within mammary gland tissue represents a promising strategy for mitigating mastitis in animals. To effectively combat this condition, it is essential to conduct comprehensive research into the mechanisms underlying oxidative stress and apoptosis in the mammary glands. Understanding these pathways will aid in developing targeted interventions aimed at reducing oxidative damage and improving overall animal health and productivity (57).

## 4 Bioactive compounds boost antioxidant and anti-inflammatory responses by activating Nrf2/KEAP1 signaling pathway to combat mastitis

**Figure 3:**
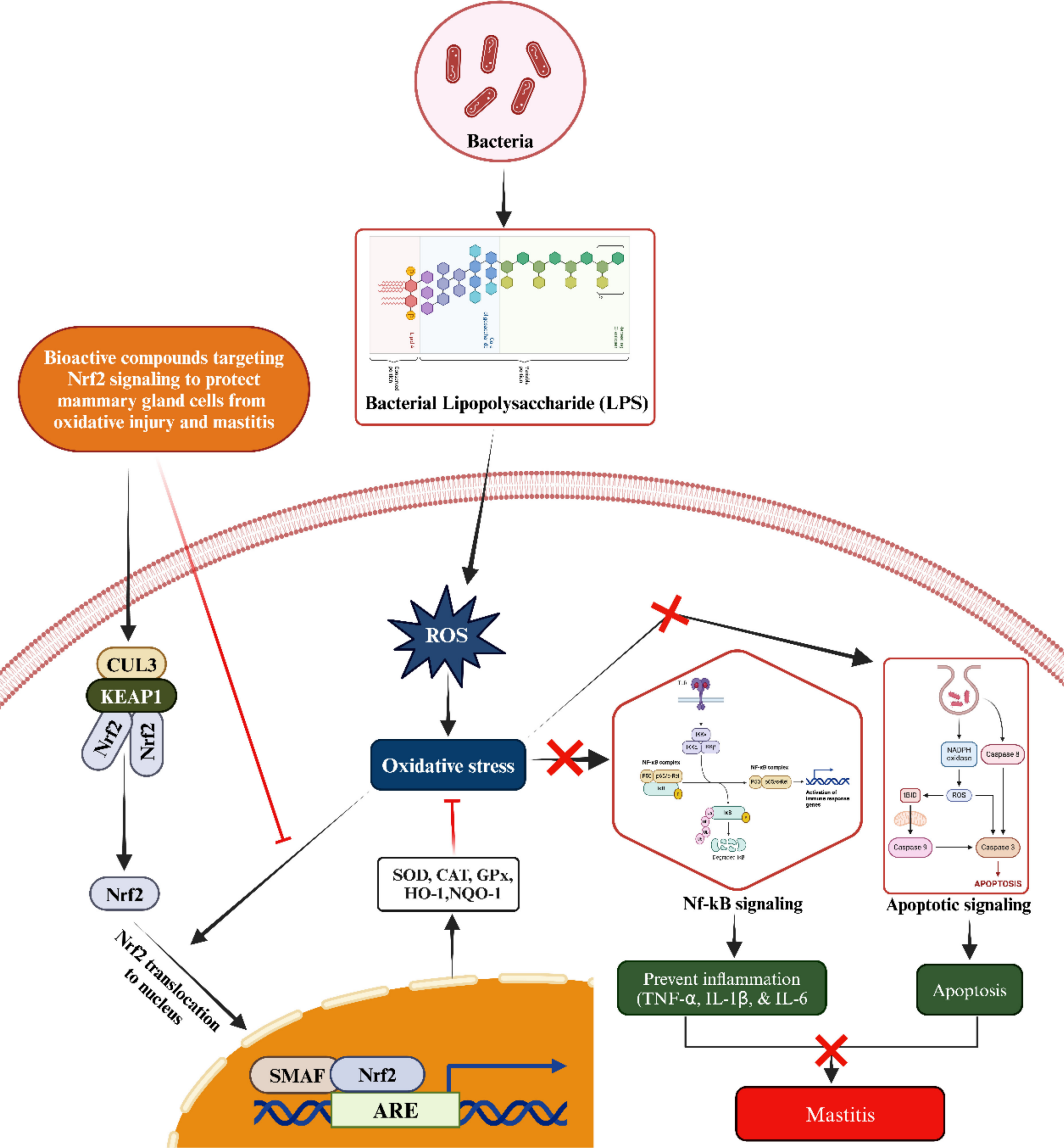
Mechanism of activating the Nrf2/KEAP1 signaling pathway to counteract oxidative stress and subsequent inflammatory reactions, including mastitis, induced by LPS through the administration of bioactive compounds from an external source. Upon administration, the bioactive compound triggers the activation of Nrf2. This activated Nrf2 then translocates to the nucleus, where it forms a heterodimer with small Maf proteins (sMaf) by binding to SMAF. This Nrf2/sMaf heterodimer specifically binds to a cis-acting enhancer known as the antioxidant response element (ARE), initiating the transcription of a range of antioxidant genes. These ARE-regulated genes play a crucial role in blocking oxidative stress and inhibiting the NF-kB signaling pathway activated by LPS. Additionally, they enhance antioxidant and anti-inflammatory responses, thereby preventing inflammatory changes in mammary epithelial cells.

Bioactive compounds interact with and inhibit the activity of KEAP1, a cytoplasmic repressor that binds to NRF2 under normal conditions. When KEAP1 is inhibited, NRF2 is released and translocates to the nucleus. Nrf2, upon translocating to the nucleus, binds to antioxidant response elements (AREs) located in the promoter regions of various genes (52). This binding activates the expression of key antioxidant genes, including heme oxygenase-1 (HO-1), superoxide dismutase (SOD), catalase (CAT), glutathione peroxidase (GPX), and NAD(P)H quinone oxidoreductase 1 (NQO1) (**Figure 3**) (58, 59). The increase in these enzymes enhances the cell’s antioxidant capacity, reducing oxidative stress by neutralizing ROS. Bioactive compounds can inhibit the activation of the NF-κB pathway by preventing the degradation of inhibitor of kappa B (IκBα), which keeps NF-κB inactive in the cytoplasm. The one possible mechanism associated with suppression might be due to the inhibition of ROS via upregulating the antioxidant status. By inhibiting the translocation of NF-κB to the nucleus, the transcription of pro-inflammatory genes (such as TNF-α, IL-1β, and IL-6) is reduced (60–63). This result in a decrease in the production of inflammatory cytokines and chemokines, leading to reduced inflammation. Considering the critical role of the Nrf2/KEAP1 signaling pathway, a large number of bioactive compounds such as phytoncide, melatonin, Chinese propolis, bergenin, and resveratrol etc., have been systematically evaluated for their regulatory effects on this pathway to alleviate mastitis in animals (64–68).

### 4.1 Bioactive compounds boost antioxidant and anti-inflammatory responses by activating Nrf2/KEAP1 signaling to combat mastitis: *in vitro* evidence

It has been well established through *in vitro* experiments that bioactive compounds can enhance the activation of the Nrf2/KEAP1 signaling pathway. This activation leads to improved antioxidant defenses and potential therapeutic benefits in mastitis. This section focuses on the specific mechanisms by which these compounds influence the Nrf2/KEAP1 signaling cascade. By providing insights into their role in combating mastitis at the cellular level, this section enhances our understanding of how these compounds work.

Consistently, the administration of *Tanshinone IIa* to LPS-stimulated cow mammary epithelial cells (CMECs) was observed to have positive effects (62). In addition, it was found that *Tanshinone IIa* reduced oxidative stress markers, restored mitochondrial function, and enhanced antioxidant enzyme activity by activating the Nrf2/Keap1 signaling pathway (62). The study conducted by Kang et al. (64) explored the effects of phytoncide extracted from pinecones on BMECs, specifically focusing on its anti-inflammatory and antioxidant properties using an *in vitro* model. To induce inflammation, the cells were treated with LPS. Their findings revealed that phytoncide significantly reduced the expression of pro-inflammatory cytokines such as TNF-α, IL-6, and IL-1β. Moreover, it inhibited the NF-κB signaling pathway (64). Furthermore, phytoncide was found to activate Nrf2 and enhance the antioxidant response in BMECs. Similarly, in a study conducted by Yu et al. (65), BMECs were pre-treated with melatonin (43 µM and 430 µM) for 12 hours prior to LPS stimulation (100 ng/mL) for an additional 12 hours to induce inflammation. Their results showed that melatonin inhibited the LPS-binding protein–CD14–TLR4 signaling pathway, leading to a decrease in pro-inflammatory mediators and an increase in anti-inflammatory responses, followed by enhanced antioxidant defenses through the activation Nrf2 pathway (65). The protective effects of Chinese propolis on BMECs against damage caused by LPS- induced mastitis. Briefly, Chinese propolis preserved cell viability in bovine mammary cells exposed to pathogens and reduced pro-inflammatory cytokine expression (IL-6, TNF-α). It also boosted antioxidant gene expression (HO-1, Txnrd-1, GCLM) and inhibited NF-κB activation while enhancing Nrf2-ARE activity, which are key pathways in inflammation and oxidative stress defense (66). In a related study, Ma X et al. (69) explored the protective effects of selenomethionine against inflammatory injury and oxidative damage in BMECs induced by *Klebsiella pneumoniae* (*K. pneumoniae*). Their findings revealed that *K. pneumoniae* suppresses the Nrf2 signaling pathway and antioxidant enzyme activity, resulting in elevated inflammatory cytokine levels and activation of the NF-κB pathway. However, pre-treatment with 4 μM selenomethionine prior to infection effectively protected BMECs by activating Nrf2 signaling and inhibiting NF-κB activation, thus mitigating both inflammation and oxidative stress (69).

A study investigated the cytoprotective effects of resveratrol on BMECs exposed to oxidative stress induced by H_2_O_2_ (67). Resveratrol pretreatment rescued cell viability, reduced intracellular ROS accumulation, and prevented endoplasmic reticulum stress and mitochondria-related apoptosis. It also upregulated the expression of multiple antioxidant defense genes (Nrf2, HO-1, TrxR-1 and xCT), playing a key role in bolstering the cells’ antioxidant mechanisms. Furthermore, they noticed that the protective effects of resveratrol were dependent on the activation of the Nrf2, with its induction mediated by the phosphoinosi-tide-3-kinase/protein kinase B (PI3K/Akt) and ERK/MAPK pathways and negatively regulated by the p38/MAPK pathway (67). Furthermore, Ma Y et al. (70) demonstrated that green tea polyphenols (GTPs) protect BMECs from inflammation, oxidative stress, and apoptosis induced by H_2_O_2_ (500 μM for 12 h). The BMECs were pre-treated with various concentrations of GTPs before being exposed to H_2_O_2_ to induce oxidative damage. It was found that GTPs treatment significantly decreased the level of MDA and increased the expressions of Nrf2, HO-1, SOD, CAT, and GSH-Px, indicating enhanced antioxidant capacity and reduced oxidative stress in BMECs (70). Moreover, Zhu et al. (71) elucidated the role of Ubiquitin-specific protease 14 (USP14) in mediating LPS-induced oxidative stress and ferroptosis, leading to the regulation of IL-6. They found that Ferrostatin-1 (Fer-1) upregulated Nrf2 levels following the suppression of oxidative stress, highlighting its potential in mitigating oxidative stress-induced damage in the goat MECs (71). Supplementation with methionine and arginine has been evidenced to ameliorate oxidative stress and inflammation provoked by LPS in BMECs (72). They administered methionine and arginine and incubated for 12 hours followed by LPS (1 μg/mL) treatment obtained from *E. coli.* These nutrients downregulated the expressions of chemokine (C-X-C motif) ligand 2 (CXCL2) and IL-1β and upregulated the levels of solute carrier family 36 member 1 (SLC36A1) and solute carrier family 7 member 1 (SLC7A1), thereby mitigating inflammatory alterations in the mammary gland. Additionally, Dai et al. (72) observed heightened levels of NFE2L2, SOD2, NQO1, and GPX1, indicative of enhanced antioxidant status following methionine and arginine supplementation. Consequently, a study has shown that LPS (1μg/mL) induced inflammatory changes such as elevated expressions of TNF-α, IL-1β, and IL-6 and heightened oxidative stress through the inhibition of Nrf2, HO-1, NQO-1, and thioredoxin reductase 1 (TXNRD1) in BMECs. However, hydroxytyrosol (10 and 25 μM) treatment prevented LPS-induced mastitis by increasing the levels of Nrf2, HO-1, NQO-1, TXNRD1, TNF-α, IL-1β, and IL-6 in mammary gland tissue (73). Similarly, Guo et al. (74) demonstrated that butyrate mitigates oxidative stress and inflammatory responses by reducing the levels of TNF-α, IL-1β, and IL-6, while enhancing the expression of SOD2, Nrf2, and AMP-activated protein kinase (AMPK) in BMECs. These actions contribute to protecting the mammary gland against LPS-induced mastitis. Additionally, vitamin A supplementation was shown to prevent LPS-induced oxidative stress by upregulating Nrf2 and GPX expression and downregulating NF-κB, IL-1, and IL-1β (75, 76).

Astragaloside IV, an extract from *Astragalus membranaceus* (Fisch) Bunge, prevented ammonia-induced oxidative stress and apoptosis by augmenting the expression of HO-1, xCT (also known as SLC7A11), and Nrf2 signaling, and suppressing Bax, caspase 3, p53, while upregulating Bcl2 levels (77). Furthermore, they elucidated that Astragaloside IV regulates Nrf2 signaling via the activation of PI3K/AKT and mitogen-activated protein kinase/extracellular signal-regulated kinase (MAPK/ERK) pathways in BMECs (77). Consequently, it has been documented that melatonin (1 mM) inhibited LPS-induced oxidative stress and inflammation in mouse mammary gland tissue (78). Furthermore, the melatonin treatment significantly downregulated the levels of TNF-α, IL-1β, IL-6, CXCL1, monocyte chemoattractant protein-1 (MCP-1), and regulated upon activation normal T-cell expressed and secreted (RANTES), enhanced Nrf2 levels, and suppressed inducible nitric oxide synthase (iNOS) and cyclooxygenase-2 (COX-2) (78). Puerarin supplementation (400 mg mixed with a standard diet daily) has been shown to significantly reduce inflammatory cytokines and somatic cell count (SCC) in the milk of cows with mastitis. Additionally, Puerarin (40 µM) treatment was found to decrease the expression of NF-κB-associated inflammatory factors (IL-6 and IL-8) while increasing the levels of Nrf2 and its associated antioxidant genes (GSH, SOD, CAT), thereby mitigating inflammation and oxidative stress induced by H_2_O_2_ (400 µM) in BMECs (79). This *in-vitro* compilation emphasizes the therapeutic potential of targeting Nrf2/KEAP1 signaling as a strategy for managing mastitis in animals. It also highlights the need for additional research in this field to fully utilize the benefits of bioactive compounds in animal health and disease management. For ease of reference, the roles of various bioactive compounds in preventing and reducing mastitis, particularly through the regulation of Nrf2/KEAP1 signaling pathway, are summarized in **Table 1**.

**Table 1:** Bioactive compounds targeting Nrf2/KEAP1/HO1 signaling pathway to combat mastitis*: In vitro* evidence.

Causative Agent	Therapeutic Agent/dosage/method of administration	Target pathway	Outcomes	Experimental model	References
Lipoteichoic Acid (100 µg/mL of LTA for 6 hours)–induced mastitis	Metformin (1,1-Dimethylbiguanide hydrochloride, derived from *Galega officinalis*) 3 mM for 12 hours prior to LTA exposure/cell culture	AMPK/Nrf2/NF-κB Signaling Pathway	⟡ Metformin activates the NRF2 pathway by affecting cellular energy status. It does this by inhibiting mitochondrial complex I, which reduces ATP production and increases the AMP/ATP ratio. This, in turn, activates AMP-activated protein kinase (AMPK), a crucial regulator of cellular energy balance. ⟡ When AMPK is activated by metformin, it can phosphorylate and activate NRF2. Additionally, metformin has been found to inhibit the activation of NF-κB, mainly through the activation of AMPK. This inhibition can prevent the phosphorylation and degradation of IκB by inhibiting IκB kinase ⟡ Collectively metformin significantly downregulated the expression of NF-κB, cyclooxygenase-2, IL-1β, and IL-6 and upregulated the levels of AMPK, Nrf2 and HO-1 to enhance antioxidant and anti-inflammatory responses in BMECs	BMECs	(35)
LPS (10 μg/mL)-induced mastitis	Chlorogenic acid (Traditional Chinese medicinal herbs such as honeysuckle, Eucommia ulmoides leaves, and chrysanthemum)/10 μg/mL/cell culture	NF-κB/Nrf2/HO-1 signaling pathway	⟡ Prevented the degradation of IκBα to inhibit the activation of NF-κB and promote the degradation of Keap1 to facilitate the release of Nrf2 ⟡ Suppressed the inflammatory changes by reducing the expressions of NF-κB, IL-6, IL-8, TNF-α, IL-1β, and iNOS ⟡ Enhanced Antioxidant responses by elevation the level of CHOP, Nrf2 and HO-1	BMECs	(57)
LPS (10 μg/mL)-induced mastitis	Tanshinone IIa (Diterpene quinone derived from the roots of *Salvia miltiorrhiza)* 2.5 μM/cell culture	Nrf2/Keap1 signaling pathway	⟡ The Tanshinone IIa Nrf2 signaling pathway potentially leads to the upregulation of antioxidant enzymes like HO-1, NQO1, and glutathione S-transferase (GST). These enzymes play a crucial role in neutralizing ROS and reducing oxidative stress, which is a significant factor in the development and progression of mastitis. ⟡ Furthermore, the activation of Nrf2 by Tanshinone IIa also results in the suppression of pro-inflammatory cytokines. This occurs because the antioxidant enzymes induced by Nrf2 can decrease oxidative stress levels, consequently reducing the activation of NF-κB, which is a major transcription factor responsible for the expression of these inflammatory cytokines. ⟡ Prevented mastitis via regulation of Keap1/Nrf2 signaling pathways	BMECs	(61)
LPS (10 µg/mL)-induced mastitis	Sodium butyrate (sodium salt of butyric acid)/2 mM/cell culture	Nrf2 signaling pathway	⟡ Upregulated the expression of Nrf2, SOD, GSH-Px, CAT, HO-1, and NQO1 suppressed the level of MDA to promote antioxidant response ⟡ Inflammatory changes were reversed by downregulating the levels of IL-6, IL-Iβ, TNF-α, NF-κB ⟡ Apoptosis was prevented by inhibiting the levels of caspases and Bax and elevated the expression of Bcl2 ⟡ By activating Nrf2, Sodium butyrate helps in reducing the production of pro-inflammatory cytokines like TNF-α, IL-1β, and IL-6. This effect is partly due to the crosstalk between Nrf2 and NF-κB pathways, where Nrf2 activation can inhibit NF-κB-mediated inflammatory signaling. ⟡ Sodium butyrate is also a well-known histone deacetylase (HDAC) inhibitor. By inhibiting HDACs, it can increase the acetylation of histones, which relaxes chromatin structure and facilitates the transcription of Nrf2 target genes	BMECs	(80)
LPS-induced mastitis	Lentinan (β-1,3-glucan)/20 μg/mL/direct addition to the cell culture medium	Nrf2 pathway	⟡ Lentinan blocked the expression of NF-κB and MAPK to relieve inflammatory changes ⟡ Enhanced Nrf2 and HO-1 level to suppress to oxidative stress and MBECs injury	BMECs	(81)
LPS-induced mastitis	2-methyl nonyl ketone (Derived from Houttuynia Cordata Thunb) 25 μg/mL/administered directly to cell culture medium	TLR4-NF-κB and Nrf2/HO-1 signaling pathway	⟡ Enhanced the expression of TLR4-NF-κB and suppressed Nrf2/HO-1 level by LPS levels. ⟡ 2-Methyl Nonyl Ketone modify Keap1, which can lead to the release of Nrf2 ⟡ Nrf2/HO-1 level was improved by 2-methyl nonyl ketone in mammary cells, and prevented inflammatory changes caused by activated NF-κB and TLR4 to inflammatory changes in the mammary gland tissue ⟡ Finally prevented cell injury from inflammation and oxidative stress	BMECs	(82)
LPS-induced mastitis	Betaine (Trimethylglycine, a product derived from *Beta vulgaris*)/25 mM/administered directly to cell culture medium	Nrf2/HO-1 signaling pathway	⟡ Promoting the dissociation of Nrf2 from Keap1 ⟡ Activated Nrf2 regulates the levels of SOD, GSH-Px, and reduced the levels of MDA, IL-1β, IL-6 and TNFα to relieve inflammatory changes and oxidative stress in BMECs via activation Nrf2/HO-1 and suppressing signaling pathways	BMECs	(83)
LPS*-*induced mastitis	Niacin (Vitamin B3/nicotinic acid)/50 mg/kg/administered intraperitoneally	AMPK/Nrf2 signaling pathway	⟡ Niacin, particularly through its role in NAD+ synthesis, supports the cellular redox state and energy metabolism. ⟡ NAD+ is a cofactor for the activity of sirtuins, which are involved in the deacetylation of proteins, including those that can influence NRF2 activity. ⟡ Activate the expression of GPR109A, which suppress the proinflammatory cytokines (TNF-α, IL-6 and IL-1β) and regulated the Nrf2 to enhance the anti-inflammatory and antioxidant responses to mitigate mastitis	MMECs	(84)
LPS (100 μg/mL)-induced mastitis	Curcumin (Diferuloylmethane, a compound derived from rhizomes of turmeric)/10 μM/administration directly into cell culture	Nrf2 signaling pathway	⟡ The electrophilic properties of the α, β-unsaturated carbonyl group in curcumin can modify cysteine residues in Keap1, leading to the disruption of the Keap1-NRF2 complex. This allows NRF2 to accumulate and translocate to the nucleus ⟡ Once activated, NRF2 increases the expression of several genes involved HO-1 and NQO-1 to improve the antioxidant activity ⟡ Apoptosis was inhibited via upregulation of Bcl2 and suppression of Bax ⟡ The inflammatory changes were prevented via downregulation of TNF-α, IL-8, IL-6 and IL-1β ⟡ Protected BMECs from oxidative damage	BMECs	(85)
LPS (50 μg/mL of LPS for 12 hours)-induced mastitis	Ferulic acid (4-hydroxy-3-methoxycinnamic acid, plant based) 15 μg/mL/administered into cell culture 2 hours before LPS treatment	NF-κB/Nrf2 signaling pathways	⟡ Antioxidant ability was enhanced through regulation of Nrf2 followed by elevated levels of its downstream genes SOD, GPX, COX2 and suppressed the expression of MDA ⟡ Reduced the level of Bax and elevated the expression of Bcl2 ⟡ Anti-inflammatory response was promoted via regulation of NF-kB, TNF-α, IL-6, and IL-1β	BMECs	(86)
LPS (12 μg/mL of LPS for 12 hours)-induced mastitis	Menthol (2-Isopropyl-5-methylcyclohexanol, a compound extracted from the essential oils of mint plants)/200 μM/administered into cell culture	AMPK/ULK1/Nrf-2/autophagy pathway	⟡ Suppressed the levels of TNF-α, IL-6, and IL-1β ⟡ Promoted the expressions of ULK1, AMPK, and Nrf2 ⟡ Restored synthesis of milk fat and milk protein	BMECs	(87)
LPS (100 ng/mL of LPS)-induced mastitis	Dandelion (medicinal plant based)/10µg/mL/applied directly to the cultured cells *in vitro*	Nrf2 signaling pathway	⟡ Dandelions contains Chicoric Acid and Beta-Carotene, which are known for their strong antioxidant effects. By scavenging free radicals and reducing oxidative stress, these compounds can help activate NRF2. ⟡ Taraxasterol and Luteolin in Dandelions by reducing inflammation, these compounds can help to modulate NRF2 signaling ⟡ Ameliorated the level ROS production and enhanced Nrf2 expression. ⟡ Protected mammary gland damage from oxidative stress	BMECs	(88)
*Streptococcus lutetiensis* induced oxidative stress and autophagy	N-Acetyl-L-cysteine (NAC)/5 Mm/cell culture	Nrf2/Keap1 signaling pathway	⟡ NAC promotes the activation and nuclear translocation of Nrf2 by modifying cysteine residues on Keap1, a protein that normally inhibits Nrf2 ⟡ NAC serves as a precursor to GSH, a critical antioxidant that neutralizes ROS and reduces oxidative stress. By replenishing GSH levels, NAC helps maintain redox balance in the mammary gland during mastitis. ⟡ NAC promotes the activation and nuclear translocation of Nrf2 by modifying cysteine residues on Keap1 ⟡ Its ability to activate Nrf2 indirectly inhibits the NF-κB signaling pathway, reducing the inflammatory response and preventing excessive tissue damage. ⟡ Enhanced the antioxidant response by elevating the level of Nrf2, HO1, and NQO1, and reduced ROS production	BMECs	(89)
γ-d-Glutamyl-meso-diaminopimelic acid induced oxidative stress and inflammation	Glutamine/0.6 mM for 12/administration directly into cell culture	NOD1/NF-κB and ERK/Nrf2 pathways	⟡ Downregulated the levels of NF-κB, NOD1, IL-6 and TNF-α by glutamine treatment and inflammatory changes were relieved ⟡ Enhanced the expression of ERK, Nrf2, SOD, CAT, NQO1 and HO-1 to improve the antioxidant response	BMECs	(90)
H_2_O_2_ (100 μμ) induced oxidative stress and inflammation	Quercetin (Plant based polyphenolic flavonoid, composed of two benzene rings (A and B) connected by a three-carbon chain that forms a closed pyran ring)/20 μμ/cell culture	MAPK/Nrf2 Signaling Pathway	⟡ Modified cysteine residues on Keap1 allow Nrf2 to escape degradation. ⟡ Alleviated oxidative stress. ⟡ Improved mice mammary epithelial cell viability and antioxidant capacity. ⟡ Restored mammary health by enhancing Nrf2, T-AOC, and MAPK expression.	MMECs	(91)
H_2_O_2_ (500 μM)-induced oxidative stress	Taurine/2.0 mM taurine for 12 h/cell culture	Nrf2-MAPK signaling pathway	⟡ Upregulated the expression on Nrf2 and inactivated the p38/MAPK pathway ⟡ Relieved the oxidative stress and guard the mammary gland tissue	PMECs	(92)
H2O2 (100 μM)-induced oxidative stress and apoptosis	Myricetin (3,5,7,3′,4′,5′-hexahydroxyflavone, a plant-based flavonoid)/5 μM/administration directly into cell culture	AMPK/Nrf2 signaling pathway	⟡ Reduced MDA and ROS level ⟡ Enhanced antioxidant response via the elevated expressions of Nrf2, T-AOC, SOD and CAT ⟡ Myricetin reduces the production of pro-inflammatory cytokines and also inhibits the activation of NF-κB	BMECs	(93)
H_2_O_2_-induced ROS production	Baicalin (5,6-Dihydroxy-4-oxoflav-2-en-7-yl β-D-glucopyranosiduronic acid, a flavonoid compound, primarily derived from the roots of ***Scutellaria baicalensis***, commonly known as Baikal skullcap or Chinese skullcap)/cell culture	Nrf2 signaling pathway	⟡ Baicalin can inhibit the Keap1-Nrf2 interaction, leading to stabilization and accumulation of Nrf2 in the cytoplasm. ⟡ Suppressed level of ROS and oxidative stress via activation of Nrf2 signaling pathway ⟡ Baicalin, through its action on Nrf2, can modulate the expression of inflammatory cytokines and other mediators, thereby contributing to a reduction in inflammation associated with mastitis	BMECs	(94)
H_2_O_2_ (500 μM)-induced oxidative stress, inflammation and apoptosis	Lycopene (Plant based carotenoid) 24 hours for 24 hours/cell culture	Nrf2/NF-κB signaling Pathway	⟡ Lycopene causes modifications in Keap1’s cysteine residues and as a result Nrf2 translocates to nucleus, and binds to antioxidant response elements (AREs) in the promoter regions of target genes. ⟡ Enhanced the antioxidant response through activation of Nrf2 ⟡ Relieved inflammation via downregulation the levels of NF-κB, TNF-α, IL-6, and IL-1β ⟡ Prevented apoptosis via upregulation of Bcl2 and decreased the expressions of Bax and caspase-3 ⟡	BMECs	(95)
H_2_O_2_ (400 µM for 24 hours)-induced Oxidative Stress and Apoptosis	Sulforaphane (1-isothiocyanato-4-methylsulfinylbutane, medicinal plant based) 5 µM/cell culture/*in vivo*	AMPK/Nrf2 Signaling Pathway	⟡ Improved antioxidant response via upregulation of Nrf2, SOD, GSH and AMPK and inhibition of MDA ⟡ Reduced apoptosis via downregulating Bax and caspase-3, and elevated the level of Bcl2 ⟡ Protected mammary epithelial cells from oxidative damage	GMECs	(96)
Deoxynivalenol (0.25 μg/mL)-induced oxidative stress and inflammatory response	Pterostilbene (4’-Methoxy-4-hydroxystilbene, derivative of resveratrol)/2.0504 μg/mL for 9 hours/cell culture	NF-κB/Nrf2/Keap1 signaling pathway	⟡ Relieved inflammatory changes via downregulation of NF-κB P65, NF-κB P50, MCP-1, COX-2, TNF-α, IL-6, and IL-1β ⟡ Enhanced Antioxidant response and inhibited ROS production by elevated levels of Nrf2, Keap1, T-AOC, SOD1, SOD2, and GSH and reduced MDA content	BMECs	(97)
Heat stress induced oxidative stress and apoptosis	Methionine/120 mg/L/cell culture	Nrf2 Signaling Pathway	⟡ Elevated the expression of Nrf2, GSH-Px, SOD, SLC7A11 and FTH1 ⟡ Inhibit the level of MDA ⟡ Met treatment further restored mitochondrial function, iron homeostasis imbalance caused by heat treatment in BMECs	BMECs	(98)
Heat (42°C)-induced oxidative stress in BMECs	Procyanidin B2/25 μM/administration directly into cell culture	Nrf2 signaling pathway	⟡ Procyanidin B2 treatment reversed the inflammatory changes and oxidative damage caused by heat stress in BMECs via activating Nrf2/HO-1 pathway	BMECs	(99)
Heat stress-induced oxidative damage	S-allyl cysteine (a natural organosulfur compound primarily derived from aged garlic (*Allium sativum*)./15 μg/mL/applied *in vitro* for 2 hours prior to the induction of heat stress.	Nrf2/HO-1 signaling pathway	⟡ Reduced ROS production, caspase-3 and Bax levels ⟡ Enhanced Nrf2, SOD, CAT, GSH-Px, HSP70, HO-1, Bcl2 expressions. ⟡ Overall enhance antioxidant efficiency and prevent apoptosis via regulation of Nrf2/HO-1 signaling pathway and prevented cell injury	BMECs	(100)
Heat stress (42°C)–induced oxidative stress and apoptosis	Betaine (Trimethylglycine, a product derived from Beta vulgaris)/25 mM/applied directly to the cultured cells *in vitro*	Nrf-2/HO-1 signaling pathway	⟡ Enhanced antioxidant activity via upregulation of SOD, CAT, HO-1, and Nrf2 and decreased MDA contents ⟡ Increased the expressions HSP70, HSP27 and Bcl2 and suppressed the Bax to relieve apoptosis	BMECs	(101)

CHOP, C/EBP Homologous Protein; MMECs, Mice mammary epithelial cells; BMECs, Bovine mammary epithelial cells; GMECs, goat mammary epithelial cells; TNF-α, necrosis factor-α; interleukin (IL)-1β and IL-6); COX2, cyclooxygenase-2; iNOS, inducible nitric oxide synthase; NF-κB, nuclear factor kappa-B; MAPK, mitogen-activated protein kinase; SLC7A11, solute carrier family 7, member 11; FTH1, ferritin heavy chain 1; PMECs, porcine mammary epithelial cells; MPO, myeloperoxidase; NLRP3, Nucleotide-binding oligomerization domain-like receptor containing pyrin domain 3; ULK1, unc-51 like kinase 1; GSK-3 beta, Glycogen synthase kinase-3 beta; MCP-1, monocyte chemotactic protein 1; NLRP3, NOD-like receptor protein 3.

### 4.2 Bioactive compounds boost antioxidant and anti-inflammatory responses by activating Nrf2/KEAP1 signaling to combat mastitis: *in vivo* evidence

Recent *in vivo* studies have demonstrated that bioactive compounds can significantly enhance antioxidant and anti-inflammatory responses by activating the Nrf2/KEAP1 signaling pathway, offering a promising therapeutic approach for combating mastitis. For example, a study conducted by Ding et al. (102) investigated the effects of Rutin supplementation on goat mammary gland tissue during the periparturient period. The researchers administered Rutin at doses of 50 and 100 mg/kg body weight per day for 28 days prior to and 28 days after parturition. The results showed significant reductions in the levels of BHB and MDA, two markers of oxidative stress, and increased expressions of Nrf2, CAT, GSH-Px, SOD, and T-AOC, indicating enhanced antioxidant activity in the mammary gland tissue. Furthermore, the study found that Rutin treatment effectively prevented apoptosis and inflammation in the mammary gland. This was evidenced by the suppression of pro-apoptotic proteins Bax, caspase-3, and caspase-9, and the elevation of the anti-apoptotic protein Bcl2. These changes in apoptotic markers contributed to the preservation of mammary gland health (102). In addition to its anti-apoptotic effects, Rutin also exhibited anti-inflammatory properties. It downregulated the expressions of the pro-inflammatory cytokine TNF-α and the transcription factor NF-κB, thus mitigating inflammatory changes in the mammary tissue of goats during the periparturient period (102). In a separate study, Lebda et al. (103) established an LPS-induced rat mastitis model and supplemented it with nanocurcumin at a dose of 35 mg/kg body weight, administered orally for a 14-day period. They found that nanocurcumin increased antioxidant activity by increasing the expressions of Nrf2 and GSH-Px and decreasing MDA levels. Additionally, nanocurcumin reduced inflammation by decreasing the expressions of TNF-α, IL-1β, TLR4, NF-κB p65, and high mobility group box 1 (HMGB1) (103). Moreover, extensive research has demonstrated that supplementation with cis-9, trans-11 conjugated linoleic acid (CLA) at a dosage of 70 g can enhance the anti-inflammatory and antioxidant responses in BMECs in response to LPS-induced inflammation and oxidative stress (104–106). Additionally, these studies have reported elevated blood glucose levels and reduced concentrations of BHB in cows receiving CLA supplementation (104–106). Additionally, it was observed that the positive effects mentioned above were a result of the upregulation of Nrf2 and the suppression of autophagy induced by ROS when CLA supplementation was introduced. This, in turn, contributed to the promotion of mammary gland health (107). Consistently a study found that sulforaphane administration to mice at a dose of 50 mg/kg/day/intraperitoneally 7 days LPS in mice. Following sulforaphane administration, to create mastitis model, LPS was injected into the mammary ducts of the mice (108). These findings were further validated *in vitro* using primary goat mammary epithelial cells (GMECs) treated with both sulforaphane (20 µM) and LPS. In both *in vivo* and *in vitro* experiments, sulforaphane significantly decreased the expression of inflammatory cytokines and the protein levels of key inflammatory mediators (101, 108). A study found that corynoline intraperitoneal injection in mice significantly reduced the expression of pro-inflammatory cytokines, such as TNF-α, IL-1β, and IL-6, in the mammary tissues of LPS (intramammary)-induced mice. Furthermore, the findings of the study showed that corynoline exerted its protective effect to enhance antioxidant response by regulating the AKT/GSK3β/Nrf2 signaling pathway (33). Furthermore, a study used *in vivo* experiments where cows were treated with rumen-bypassed niacin (30g/day), and *in vitro* studies using primary BMECs (34). They documented that niacin reduced somatic cell counts (SCCs) and inflammatory markers (IL-6, IL-1β, TNF-α) in both blood and milk of mastitis infected cows. Niacin activated the GPR109A receptor, phosphorylated AMPK, and promoted NRF-2 nuclear import, ultimately reducing inflammation through enhanced autophagy (34). Another study demonstrated the effectiveness of resveratrol in reducing the inflammatory response and oxidative damage caused by *S. uberis* infection in mice mammary gland tissues and both *in vitro* and *in vivo* trials supported these findings (36). The study also revealed that resveratrol activates the Nrf2 signaling pathway, which is responsible for regulating cellular antioxidant responses. Additionally, resveratrol was found to promote the degradation of Keap1 through p62 activation. This, in turn, led to increased expression of Nrf2 and its downstream antioxidant pathways (36). Therefore, it can be concluded that resveratrol’s activation of the p62-Keap1/Nrf2 signaling pathway successfully reduces oxidative damage and inflammation caused by *S. uberis* infection. Consistently, another study reported that LPS (10 μg/mL)-induced mastitis in mouse model was effectively treated with Caffeic acid at a dosage of 10 mg/kg administered intramammarily. This treatment modulated the NF-κB/Nrf2 signaling pathway, significantly reducing LPS-induced ROS production, which drives inflammatory changes and oxidative stress in mammary gland epithelial cells. Caffeic acid prevented the activation of NF-κB by activating IκBα and promoted the dissociation of Nrf2 from its cytoplasmic inhibitor Keap1 (47). By elevating Nrf2 levels and suppressing NF-κB activity, caffeic acid enhanced the antioxidant response, alleviated inflammation, and mitigated damage to mammary tissue. Furthermore, it inhibited the oxidative burst and neutrophil chemotaxis, demonstrating protective effects in MMECs (47). In a study on LPS-induced mastitis (100 µg/intramammary), Wogonin, a flavonoid derived from medicinal plants (also known as 5,7-dihydroxy-8-methoxyflavone), was administered intraperitoneally at a dosage of 40 mg/kg. They found that Wogonin treatment by targeting NF-κB/Nrf2/HO-1 signaling pathway, significantly inhibited of inflammation by reducing the expression of NF-κB, TNF-α, and IL-1β. Moreover, it enhanced the antioxidant response by increasing levels of Nrf2, HO-1, GSH, and SOD, while simultaneously decreasing MDA levels in MMECs (108). All of the studies that reported the *in vivo* effects of bioactive compounds in the treatment of mastitis by targeting Nrf2 signaling pathway have been summarized in **Table 2**.

**Table 2:** Bioactive compounds targeting Nrf2/KEAP1 signaling pathway to combat mastitis: *in vivo* evidence.

Causative Agent	Therapeutic Agent/dosage/method of administration	Target pathway	Outcomes	Experimental model	References
LPS (100 μg/intramammary)-induced mastitis	Corynoline (Benzylisoquinoline, extracted from *Corydalis bungeana* Turcz)/60 mg/kg/intraperitoneally	AKT/GSK3β/Nrf2 signaling pathway	⟡ Inhibited inflammatory changes via downregulation of NF- kB, TNF-α and IL-1β expressions ⟡ Upregulated the expressions of Nrf2, AKT and GSK3β and reduced the level of MDA ⟡ Finally, the Corynoline ameliorated mastitis by promoting antioxidant activity and anti-inflammatory response via AKT/GSK3β/Nrf2 signaling pathway	Mouse	(33)
LPS (100 μg/intramammary)-induced mastitis	Niacin (Vitamin B3/nicotinic acid)/24 g/day/orally	GPR109A/AMPK/NRF2	⟡ Elevated levels of GPR109A, Nrf2 and AMPK to enhanced antioxidant response and relieve oxidative stressed ⟡ Ameliorated the inflammatory changes via downregulating IL-6, IL-1β, and TNF-α ⟡ Prevented mastitis via regulating the GPR109A/AMPK/NRF-2 in mammary epithelial cells	Mouse	(34)
*Streptococcus uberis* (1 × 10^7^ CFU/intramammary injection)*-*induced mastitis	Resveratrol (3,5,4’-trihydroxy-trans-stilbene, medicinal plant based) 100 mg/kg/orally	Nrf2/Keap1 signaling pathway	⟡ Suppressed via inflammation and oxidative stress via activation of Nrf2/Keap1 signaling pathway ⟡ Protected mouse mammary gland from oxidative damage and mastitis	Mouse	(36)
LPS (10 μg/mL)-induced mastitis	Caffeic acid (Hydroxycinnamic acid, composed of A carboxylic acid group attached to phenyl ring through a two-carbon alkene chain) 10 mg/kg/intramammary administration	NF-κB/Nrf2 signaling pathway	⟡ Significantly reduced LPS-induced ROS production which promote the inflammatory changes and oxidative stress in mammary gland epithelial cells ⟡ Prevented the activation of NF-κB by activating IκBα and promote the dissociation of Nrf2 from its cytoplasmic inhibitor Keap1 ⟡ By elevating level of Nrf2 and suppression of NF-κB activity, caffeic acid enhanced the antioxidant response and relieved inflammation ⟡ Alleviated mammary tissue damage and inhibited the oxidative burst and neutrophil chemotaxis	Mouse	(47)
LPS-induced mastitis *in vivo* and *in vitro*	Sulforaphane (1-isothiocyanato-4-methylsulfinylbutane, medicinal plant based) 50 mg/kg/day via intraperitoneal injection for 7 days (*in vivo*) and 20 µM/cell culture (*in vitro*)	Nrf2 Signaling Pathway	⟡ Suppressed ROS level and enhanced antioxidant response ⟡ Prevented inflammatory changes by downregulating the expression levels of TNF-α, IL-1β, IL-6, COX2, iNOS, and NF-κB ⟡ Up-regulated the expression level of Nrf2	Mouse and GMECs	(108)
LPS (100 µg/intramammary)-induced mastitis	Wogonin (5,7-dihydroxy-8-methoxyflavone, medicinal plant-based flavonoid) 40 mg/kg/intraperitoneally	NF-κB/Nrf2/HO-1 signaling pathway	⟡ Inhibited inflammation (downregulated the NF-κB, TNF-α and IL-1β levels) and enhanced the antioxidant response (increased the Nrf2, HO-1, GSH, SOD and decreased MDA levels)	Mouse	(109)
LPS (2.5 mg/kg)-induced inflammation and oxidative stress	Resveratrol (3,5,4’-trihydroxy-trans-stilbene) 2 mg/kg/orally for 15 days	NF-κb/Nrf2 Signaling	⟡ Upregulated the level of Nrf2 by inhibiting oxidative stress via enhancing antioxidant response ⟡ Enhanced the levels of Nrf2 and T-AOC and combat oxidative stress ⟡ By activating Nrf2, resveratrol can inhibit the production of pro-inflammatory cytokines (e.g., TNF-α, IL-6) and reduce the expression of inflammatory mediators like NF-κB.	Mouse	(110)
LPS-induced oxidative stress	Sanguinarine (A benzophenanthridine alkaloid derived from *Sanguinaria canadensis* and *poppy Fumaria* species)/50 µM/intraperitoneally	Nrf2/HO-1 signaling pathway	⟡ Sanguinarine modifies Keap1, leading to the release of NRF2. ⟡ Enhanced the level of Nrf2 followed by elevated antioxidant response ⟡ Suppressed TNF-α and IL-1β expression followed by inhibition of inflammatory changes in MMECs	Mouse	(111)
LPS- induced mastitis	Kynurenic acid (2-Hydroxy-3-carboxy-6-methoxybenzeneacetic acid, a metabolite of tryptophan)/100 mg/kg/intraperitoneally	NF-κB/Nrf2/HO-1 signaling pathway	⟡ NF-κB, TNF-α and IL-1β mRNA expressions were inhibited ⟡ Blood-milk barrier integrity was protected from oxidative stress damage induced by LPS via activating Nrf2/HO-1 signaling pathway	Mouse	(112)
LPS (10 *μ*g)-induced mastitis	Dioscin (a natural compound extracted from the tubers of *Dioscorea japonica*)/45 mg/kg/day/orally	AMPK/Nrf2/NF-κB signaling Pathway	⟡ Promoted the expressions of AMPK and Nrf2 and ameliorated oxidative stress ⟡ Inhibited the levels of NLRP3, IL-6, IL-1β, TNF-α, and NF-κB to relieve inflammatory changes ⟡ Prevented mastitis via activation of AMPK/Nrf2/NF-κB signaling Pathway	Mouse	(113)
LPS-induced mastitis	Schisandrin A (dibenzocyclooctadiene lignane, derived from the plant *Schisandra chinensis*)/40 mg/kg/administered via intraperitoneal injection	Nrf2 signaling pathway	⟡ Enhanced antioxidant and anti-inflammatory response via activated AMPK/ULK1/Nrf2 signaling pathway ⟡ Protected mammary gland from oxidative injury and mitigated mastitis	Mouse	(114)
LPS (100 μL)-induced mastitis via nipple duct injection in 5–7 days postpartum mice	Dimethyl itaconate (Cell-permeable derivative of itaconate and a metabolite of the tricarboxylic acid cycle)/25 mg/kg/intraperitoneal	MAPKs/Nrf2/NF-κB signaling pathways	⟡ Activates Nrf2 via alkylation of KEAP1. ⟡ Reduced inflammatory changes by downregulating the levels of TLR4, NF-κB, TNF-α and IL-1β. ⟡ Relieved oxidative response and enhance antioxidant response via regulation of MAPK and Nrf2 ⟡ Overall, dimethyl itaconate prevented mastitis via activation of MAPKs/Nrf2 and inhibition of NF-κB signaling pathways	Mouse	(115)
*S. uberis* (1 × 10^7^ CFU/intramammary injection)-induced mastitis	Taurine (2-aminoethanesulfonic acid) 100 mg/kg body weight/intraperitoneally	NF-κB/AMPK/Nrf2 signaling pathway	⟡ Decreased the expressions of TLR2, CXCL2, MAPK and NF-κB to relieve the inflammation ⟡ Regulated the expression of AMPK/Nrf2 to enhance the antioxidant response ⟡ Protected mammary tissue from oxidative damage	Murine mammary glands	(116)
*S. aureus* (1 × 10^7^ CFU)*-*induced mastitis	Diosmetin (3′,5,7-trihydroxy-4′-methoxyflavone, plant based)/25 mg/kg/intramammary one hour before S. aureus treatment	NF-κb/Nrf2/HO-1 Signaling pathway	⟡ Promoted the dissociation of Nrf2 from Keap1. ⟡ Upregulated the expressions of SIRT1, GPX4, HO-1 and Nrf2 and downregulated the level of MDA and promoted the antioxidant response ⟡ Suppressed the level of MPO, TNF-α and IL-1β, and NF-kB and alleviated the inflammation	Mouse	(117)
*S. aureus* (1 × 10^7^ CFU)*-*induced oxidative stress and apoptosis	Saikosaponin (Triterpene saponin, derived from the roots of *Bupleurum falcatum*)/20 mg/kg/intramammary	SIRT1/Nrf2 Signaling pathway	⟡ Disrupted the interaction between Nrf2 and Keap1. This disruption leads to the stabilization and nuclear translocation of Nrf2. ⟡ Enhanced the level of SIRT1, Nrf2, HO-1 and GPX4 to promote antioxidant response ⟡ Suppressed the level of MPO, TNF-α and IL-1β, and NF-kB and alleviated the inflammation	Mouse	(118)

## 5 Limitations and future directions

Most of the evidence presented is based on preclinical studies involving cell cultures and animal models. However, there is a lack of clinical trials in actual dairy herds, which limits the direct applicability of these findings to real-world farming practices. The transition from laboratory research to practical applications in dairy farming remains underexplored. To translate preclinical findings into practical applications, it is essential to conduct well-designed clinical trials and field studies in dairy herds. These studies should assess the effectiveness, safety, and economic viability of bioactive compounds in preventing and treating mastitis in real-world settings.

The review emphasizes the beneficial effects of bioactive compounds in reducing oxidative stress and inflammation. However, it does not fully address the potential unintended effects, such as toxicity or interference with other cellular pathways. These aspects require careful consideration, especially in long-term or high-dose applications.

Several studies have shown that Nrf2 signaling plays a complex and multifaceted role in cancer development and progression (119–122). When Nrf2 is overactivated, it can enhance antioxidant responses. While this is beneficial under normal circumstances, it may inadvertently support tumor growth and resistance to therapy by promoting cellular survival pathways. On the other hand, some research suggests that Nrf2 could be a potential target for cancer treatment, indicating that regulating its activity could suppress tumor progression (123). These findings highlight the dual nature of Nrf2 in cancer biology. Given these insights, it is crucial to carefully assess the biological impact of Nrf2, particularly its overactivation, in future research on mastitis mitigation. Understanding the potential negative effects of Nrf2 overactivation will be essential to prevent unintended consequences and ensure the effectiveness of therapeutic interventions.

Future studies should explore the potential synergistic effects of combining multiple bioactive compounds or integrating them with existing therapeutic strategies. Such combinations might enhance efficacy and reduce the likelihood of resistance or side effects. Investigating the long-term impact and safety of bioactive compound supplementation in animals is crucial. These studies should consider potential off-target effects, the impact on milk quality and yield, and overall animal health and welfare. Beyond the biological effects, future research should assess the economic feasibility of using bioactive compounds on a large scale in dairy farming. Additionally, the environmental impact of their use, including any potential residues in milk and their effects on ecosystems, should be thoroughly evaluated.

## 6 Conclusion

In conclusion, the review highlights the pivotal role of the Nrf2/KEAP1 signaling pathway in combating mastitis through the regulation of antioxidant and anti-inflammatory responses. The evidence underscores the therapeutic potential of bioactive compounds, which activate Nrf2/KEAP1 signaling pathway, in enhancing antioxidant defenses, reducing inflammation, and mitigating cellular damage in mammary tissues. These compounds offer promising avenues for improving the health of dairy animals, particularly in the context of mastitis management. However, despite the significant progress in understanding the molecular mechanisms by which these bioactive compounds exert their effects, further research is needed to optimize their use in practical settings. Future studies should focus on combination strategies of these compounds to maximize their efficacy in preventing and treating mastitis. Moreover, the exploration of additional bioactive compounds and their interactions with other cellular pathways could provide deeper insights into their broader applications in animal health. Finally, to translate the findings from preclinical research into practical applications, it is crucial to carry out meticulously designed clinical trials and field studies within dairy herds in future.

## References

[1] Khan MZ Huang B Kou X Chen Y Liang H Ullah Q. Enhancing bovine immune, antioxidant and anti-inflammatory responses with vitamins, rumen-protected amino acids, and trace minerals to prevent periparturient mastitis. Front Immunol. (2024) 14:1290044. doi: 10.3389/fimmu.2023.1290044 38259482 PMC10800369

[2] Corrêa DC Nunes GT Barcelos RA Dos Santos JR Vogel FS Cargnelutti JF. Economic losses caused by mastitis and the influence of climate variation on the occurrence of the disease in a dairy cattle farm in southern Brazil. Trop Anim Health Production. (2024) 56:1–9. doi: 10.1007/s11250-024-03914-2 38351405

[3] Kour S Sharma N NB Kumar P Soodan JS Santos MV Son YO. Advances in diagnostic approaches and therapeutic management in bovine mastitis. Veterinary Sci. (2023) 10:449. doi: 10.3390/vetsci10070449 PMC10384116 37505854

[4] Richardet M Solari HG Cabrera VE Vissio C Agüero D Bartolomé JA. The economic evaluation of mastitis control strategies in holstein-friesian dairy herds. Animals. (2023) 13:1701. doi: 10.3390/ani13101701 37238131 PMC10215626

[5] Samaraweera AM van der Werf JH Boerner V Hermesch S. Economic values for production, fertility and mastitis traits for temperate dairy cattle breeds in tropical Sri Lanka. J Anim Breed Genet. (2022) 139:330–41. doi: 10.1111/jbg.v139.3 PMC9306856 35072970

[6] Hogeveen H Steeneveld W Wolf CA. Production diseases reduce the efficiency of dairy production: a review of the results, methods, and approaches regarding the economics of mastitis. Annu Rev Resour Econ. (2019) 11:289–312. doi: 10.1146/annurev-resource-100518-093954

[7] Puerto MA Shepley E Cue RI Warner D Dubuc J Vasseur E. The hidden cost of disease: I. Impact of the first incidence of mastitis on production and economic indicators of primiparous dairy cows. J dairy science. (2021) 104:7932–43. doi: 10.3168/jds.2020-19584 33865582

[8] Rollin E Dhuyvetter KC Overton MW. The cost of clinical mastitis in the first 30 days of lactation: An economic modeling tool. Prev veterinary Med. (2015) 122:257–64. doi: 10.1016/j.prevetmed.2015.11.006 26596651

[9] Wang L Yang F Wei XJ Luo YJ Guo WZ Zhou XZ. Prevalence and risk factors of subclinical mastitis in lactating cows in Northwest China. Israel J Veterinary Med. (2019) 74:17–22.

[10] De Vliegher S Ohnstad I Piepers S. Management and prevention of mastitis: A multifactorial approach with a focus on milking, bedding and data-management. J Integr Agriculture. (2018) 17:1214–33. doi: 10.1016/S2095-3119(17)61893-8

[11] Cobirka M Tancin V Slama P. Epidemiology and classification of mastitis. Animals. (2020) 10:2212. doi: 10.3390/ani10122212 33255907 PMC7760962

[12] Khan MZ Wang J Ma Y Chen T Ma M Ullah Q. Genetic polymorphisms in immune-and inflammation-associated genes and their association with bovine mastitis resistance/susceptibility. Front Immunol. (2023) 14:1082144. doi: 10.3389/fimmu.2023.1082144 36911690 PMC9997099

[13] Khan MZ Khan A Xiao J Ma J Ma Y Chen T. Overview of research development on the role of NF-κB signaling in mastitis. Animals. (2020) 10:1625. doi: 10.3390/ani10091625 32927884 PMC7552152

[14] Khan MZ Khan A Xiao J Ma Y Ma J Gao J. Role of the JAK-STAT pathway in bovine mastitis and milk production. Animals. (2020) 10:2107. doi: 10.3390/ani10112107 33202860 PMC7697124

[15] Khan MZ Dari G Khan A Yu Y. Genetic polymorphisms of TRAPPC9 and CD4 genes and their association with milk production and mastitis resistance phenotypic traits in Chinese Holstein. Front Veterinary Science. (2022) 9:1008497. doi: 10.3389/fvets.2022.1008497 PMC9540853 36213405

[16] Wang D Wei Y Shi L Khan MZ Fan L Wang Y. Genome-wide DNA methylation pattern in a mouse model reveals two novel genes associated with Staphylococcus aureus mastitis. Asian-Australasian J Anim Sci. (2020) 33:203. doi: 10.5713/ajas.18.0858 PMC6946959 31010979

[17] Khan MZ Wang D Liu L Usman T Wen H Zhang R. Significant genetic effects of JAK2 and DGAT1 mutations on milk fat content and mastitis resistance in Holsteins. J Dairy Res. (2019) 86:388–93. doi: 10.1017/S0022029919000682 31779717

[18] Tommasoni C Fiore E Lisuzzo A Gianesella M. Mastitis in dairy cattle: On-farm diagnostics and future perspectives. Animals. (2023) 13:2538. doi: 10.3390/ani13152538 37570346 PMC10417731

[19] Khan MZ Khan A Xiao J Dou J Liu L Yu Y. Overview of folic acid supplementation alone or in combination with vitamin B12 in dairy cattle during periparturient period. Metabolites. (2020) 10:263. doi: 10.3390/metabo10060263 32630405 PMC7344520

[20] Khan MZ Zhang Z Liu L Wang D Mi S Liu X. Folic acid supplementation regulates key immunity-associated genes and pathways during the periparturient period in dairy cows. Asian-Australasian J Anim Sci. (2020) 33:1507. doi: 10.5713/ajas.18.0852 PMC7468170 31010964

[21] Khan MZ Ma Y Xiao J Chen T Ma J Liu S. Role of selenium and vitamins E and B9 in the alleviation of bovine mastitis during the periparturient period. Antioxidants. (2022) 11:657. doi: 10.3390/antiox11040657 35453342 PMC9032172

[22] Xiao J Khan MZ Ma Y Alugongo GM Ma J Chen T. The antioxidant properties of selenium and vitamin E; their role in periparturient dairy cattle health regulation. Antioxidants. (2021) 10:1555. doi: 10.3390/antiox10101555 34679690 PMC8532922

[23] Ster C Loiselle MC Lacasse P. Effect of postcalving serum nonesterified fatty acids concentration on the functionality of bovine immune cells. J Dairy Sci. (2012) 95:708–17. doi: 10.3168/jds.2011-4695 22281335

[24] Ospina PA McArt JA Overton TR Stokol T Nydam DV. Using nonesterified fatty acids and β-hydroxybutyrate concentrations during the transition period for herd-level monitoring of increased risk of disease and decreased reproductive and milking performance. Vet Clin N Am Food Anim. Pract. (2013) 29:387–412. doi: 10.1016/j.cvfa.2013.04.003 23809897

[25] Song Y Loor JJ Li C Liang Y Li N Shu X. Enhanced mitochondrial dysfunction and oxidative stress in the mammary gland of cows with clinical ketosis. J Dairy Science. (2021) 104:6909–18. doi: 10.3168/jds.2020-19964 33715853

[26] Shi Z Li XB Peng ZC Fu SP Zhao CX Du XL. Berberine protects against NEFA-induced impairment of mitochondrial respiratory chain function and insulin signaling in bovine hepatocytes. Int J Mol Sci. (2018) 19:1691. doi: 10.3390/ijms19061691 29882814 PMC6032402

[27] Khan MZ Liu S Ma Y Ma M Ullah Q Khan IM. Overview of the effect of rumen-protected limiting amino acids (methionine and lysine) and choline on the immunity, antioxidative, and inflammatory status of periparturient ruminants. Front Immunol. (2023) 13:1042895. doi: 10.3389/fimmu.2022.1042895 36713436 PMC9878850

[28] Khan MZ Khan A Chen W Chai W Wang C. Advancements in genetic biomarkers and exogenous antioxidant supplementation for safeguarding mammalian cells against heat-induced oxidative stress and apoptosis. Antioxidants. (2024) 13:258. doi: 10.3390/antiox13030258 38539792 PMC10967571

[29] Abeyta MA Al-Qaisi M Horst EA Mayorga EJ Rodriguez-Jimenez S Goetz BM. Effects of dietary antioxidant supplementation on metabolism and inflammatory biomarkers in heat-stressed dairy cows. J Dairy Science. (2023) 106:1441–52. doi: 10.3168/jds.2022-22338 36543647

[30] Vašková J Klepcová Z Špaková I Urdzík P Štofilová J Bertková I. The importance of natural antioxidants in female reproduction. Antioxidants. (2023) 12:907. doi: 10.3390/antiox12040907 37107282 PMC10135990

[31] Meli R Monnolo A Annunziata C Pirozzi C Ferrante MC. Oxidative stress and BPA toxicity: an antioxidant approach for male and female reproductive dysfunction. Antioxidants. (2020) 9:405. doi: 10.3390/antiox9050405 32397641 PMC7278868

[32] Mohd Mutalip SS Ab-Rahim S Rajikin MH. Vitamin E as an antioxidant in female reproductive health. Antioxidants. (2018) 7:22. doi: 10.3390/antiox7020022 29373543 PMC5836012

[33] Wu Y He T Fu Y Chen J. Corynoline protects lipopolysaccharide-induced mastitis through regulating AKT/GSK3β/Nrf2 signaling pathway. Environ Toxicology. (2021) 36:2493–9. doi: 10.1002/tox.v36.12 34477289

[34] Guo W Liu J Li W Ma H Gong Q Kan X. Niacin alleviates dairy cow mastitis by regulating the GPR109A/AMPK/NRF2 signaling pathway. Int J Mol Sci. (2020) 21:3321. doi: 10.3390/ijms21093321 32397071 PMC7246865

[35] Arbab AA Lu X Abdalla IM Idris AA Chen Z Li M. Metformin inhibits lipoteichoic acid–induced oxidative stress and inflammation through AMPK/NRF2/NF-κB signaling pathway in bovine mammary epithelial cells. Front Veterinary Science. (2021) 8:661380. doi: 10.3389/fvets.2021.661380 PMC8274905 34262962

[36] Zhou Y Lan R Xu Y Zhou Y Lin X Miao J. Resveratrol alleviates oxidative stress caused by Streptococcus uberis infection via activating the Nrf2 signaling pathway. Int Immunopharmacology. (2020) 89:107076. doi: 10.1016/j.intimp.2020.107076 33045565

[37] Silwal P Kim JK Kim YJ Jo EK. Mitochondrial reactive oxygen species: double-edged weapon in host defense and pathological inflammation during infection. Front Immunol. (2020) 11:554462. doi: 10.3389/fimmu.2020.01649 PMC7457135 32922385

[38] Mirza Z Walhout AJ Ambros V. A bacterial pathogen induces developmental slowing by high reactive oxygen species and mitochondrial dysfunction in Caenorhabditis elegans. Cell Rep. (2023) 42:113189. doi: 10.1016/j.celrep.2023.113189 37801396 PMC10929622

[39] Srithanasuwan A Tata L Tananupak W Jaraja W Suriyasathaporn W Chuammitri P. Exploring the distinct immunological reactions of bovine neutrophils towards major and minor pathogens responsible for mastitis. Int J Veterinary Sci Med. (2023) 11:106–20. doi: 10.1080/23144599.2023.2262250 PMC10569347 37841527

[40] Rainard P Foucras G Martins RP. Adaptive cell-mediated immunity in the mammary gland of dairy ruminants. Front Veterinary Science. (2022) 9:854890. doi: 10.3389/fvets.2022.854890 PMC9019600 35464360

[41] Chen Y Yang J Huang Z Yin B Umar T Yang C. Vitexin mitigates Staphylococcus aureus-induced mastitis via regulation of ROS/ER stress/NF-κB/MAPK pathway. Oxid Med Cell Longevity. (2022) 2022:7977433. doi: 10.1155/2022/7977433 PMC9252844 35795861

[42] Ma F Yang S Zhou M Lu Y Deng B Zhang J. NADPH oxidase-derived reactive oxygen species production activates the ERK1/2 pathway in neutrophil extracellular traps formation by Streptococcus agalactiae isolated from clinical mastitis bovine. Veterinary Microbiol. (2022) 268:109427. doi: 10.1016/j.vetmic.2022.109427 35405476

[43] Li B Wan Z Wang Z Zuo J Xu Y Han X. TLR2 signaling pathway combats Streptococcus uberis infection by inducing mitochondrial reactive oxygen species production. Cells. (2020) 9:494. doi: 10.3390/cells9020494 32098158 PMC7072855

[44] Biswas S Mukherjee R Chakravarti S Bera AK Bandyopadhyay S De UK. Influence of pathogens specific subclinical mastitis on oxidative status and mineral metabolism of yak. Emerging Anim Species. (2023) 8:100028. doi: 10.1016/j.eas.2023.100028

[45] Ma Y Cheng L Gao X Elsabagh M Feng Y Li Z. Melatonin modulates lipopolysaccharides-induced inflammatory response and maintains circadian rhythm associated with histone H3 acetylation in bovine mammary epithelial cells. J Funct Foods. (2024) 116:106156. doi: 10.1016/j.jff.2024.106156

[46] Cai X Zhou Z Kan X Xu P Guo W Fu S. Daidzein relieves lipopolysaccharide-induced mastitis through inhibiting MAPKs and AKT/NF-κB P65 signaling pathways. Rev Bras Farmacognosia. (2024) 29:1–2. doi: 10.1007/s43450-024-00529-4

[47] Yu C Zhang C Huai Y Liu D Zhang M Wang H. The inhibition effect of caffeic acid on NOX/ROS-dependent macrophages M1-like polarization contributes to relieve the LPS-induced mice mastitis. Cytokine. (2024) 174:156471. doi: 10.1016/j.cyto.2023.156471 38103301

[48] Luo H Li Y Xie J Xu C Zhang Z Li M. Effect and mechanism of Prunella vulgaris L. extract on alleviating lipopolysaccharide-induced acute mastitis in protecting the blood-milk barrier and reducing inflammation. J Ethnopharmacology. (2024) 12:117998. doi: 10.1016/j.jep.2024.117998 38484956

[49] Choudhary RK Olszanski L McFadden TB Lalonde C Spitzer A Shangraw EM. Systemic and local responses of cytokines and tissue histology following intramammary lipopolysaccharide challenge in dairy cows. J Dairy Science. (2024) 107:1299–310. doi: 10.3168/jds.2023-23543 37777007

[50] Abuelo A Hernández J Benedito JL Castillo C. Redox biology in transition periods of dairy cattle: Role in the health of periparturient and neonatal animals. Antioxidants. (2019) 8:20. doi: 10.3390/antiox8010020 30642108 PMC6356809

[51] Aitken SL Karcher EL Rezamand P Gandy JC VandeHaar MJ Capuco AV. Evaluation of antioxidant and proinflammatory gene expression in bovine mammary tissue during the periparturient period. J dairy science. (2009) 92:589–98. doi: 10.3168/jds.2008-1551 19164669

[52] Huang Q Liu J Peng C Han X Tan Z. Hesperidin ameliorates H2O2-induced bovine mammary epithelial cell oxidative stress via the Nrf2 signaling pathway. J Anim Sci Biotechnol. (2024) 15:57. doi: 10.1186/s40104-024-01012-9 38589950 PMC11003082

[53] Senthamilan S Aggarwal A Grewal S Rani S Vats P Pal P. Pre-treatment but not co-treatment with vitexin alleviates hyperthermia induced oxidative stress and inflammation in buffalo mammary epithelial cells. J Reprod Immunol. (2023) 158:103979. doi: 10.1016/j.jri.2023.103979 37348446

[54] Ayemele AG Tilahun M Lingling S Elsaadawy SA Guo Z Zhao G. Oxidative stress in dairy cows: insights into the mechanistic mode of actions and mitigating strategies. Antioxidants. (2021) 10:1918. doi: 10.3390/antiox10121918 34943022 PMC8750585

[55] Rakib MR Zhou M Xu S Liu Y Khan MA Han B. Effect of heat stress on udder health of dairy cows. J dairy Res. (2020) 87:315–21. doi: 10.1017/S0022029920000886

[56] Zhu H Cao W Huang Y Karrow NA Yang Z. Involvement of pyocyanin in promoting LPS-induced apoptosis, inflammation, and oxidative stress in bovine mammary epithelium cells. Agriculture. (2023) 13:2192. doi: 10.3390/agriculture13122192

[57] Liu D Lin J He W Huang K. Selenium and taurine combination is better than alone in protecting lipopolysaccharide-induced mammary inflammatory lesions via activating PI3K/Akt/mTOR signaling pathway by scavenging intracellular ROS. Oxid Med Cell Longevity. (2021) 2021:5048375. doi: 10.1155/2021/5048375 PMC8687852 34938382

[58] Lyu CC Ji XY Che HY Meng Y Wu HY Zhang JB. CGA alleviates LPS-induced inflammation and milk fat reduction in BMECs through the NF-κB signaling pathway. Heliyon. (2024) 10:e25004. doi: 10.1016/j.heliyon.2024.e25004 38317876 PMC10838784

[59] Zhou M Barkema HW Gao J Yang J Wang Y Kastelic JP. MicroRNA miR-223 modulates NLRP3 and Keap1, mitigating lipopolysaccharide-induced inflammation and oxidative stress in bovine mammary epithelial cells and murine mammary glands. Veterinary Res. (2023) 1454:78. doi: 10.1186/s13567-023-01206-5 PMC10503159 37710276

[60] Zhao W Deng Z Barkema HW Xu M Gao J Liu G. Nrf2 and NF-κB/NLRP3 inflammasome pathways are involved in Prototheca bovis infections of mouse mammary gland tissue and mammary epithelial cells. Free Radical Biol Med. (2022) 1184:148–57. doi: 10.1016/j.freeradbiomed.2022.04.005 35417750

[61] Ko YC Choi HS Kim SL Yun BS Lee DS. Anti-inflammatory effects of (9Z, 11E)-13-Oxooctadeca-9, 11-dienoic acid (13-KODE) derived from Salicornia herbacea L. @ on lipopolysaccharide-stimulated murine macrophage via NF-kB and MAPK inhibition and Nrf2/HO-1 signaling activation. Antioxidants. (2022) 11:180. doi: 10.3390/antiox11020180 35204063 PMC8868157

[62] Fu K Sun Y Wang J Cao R. Tanshinone IIa alleviates LPS-induced oxidative stress in dairy cow mammary epithelial cells by activating the Nrf2 signalling pathway. Res Veterinary Science. (2022) 151:149–55. doi: 10.1016/j.rvsc.2022.08.008 36027684

[63] Khan MZ Li L Wang T Liu X Chen W Ma Q. Bioactive compounds and probiotics mitigate mastitis by targeting NF-κB signaling pathway. Biomolecules. (2024) 14:1011. doi: 10.3390/biom14081011 39199398 PMC11352841

[64] Kang S Lee JS Lee HC Petriello MC Kim BY Do JT. Phytoncide extracted from pinecone decreases LPS-induced inflammatory responses in bovine mammary epithelial cells. J Microbiol Biotechnol. (2017) 26:579–87. doi: 10.4014/jmb.1510.10070 26608166

[65] Yu GM Kubota H Okita M Maeda T. The anti-inflammatory and antioxidant effects of melatonin on LPS-stimulated bovine mammary epithelial cells. PloS One. (2017) 12:e0178525. doi: 10.1371/journal.pone.0178525 28542575 PMC5444821

[66] Wang K Jin XL Shen XG Sun LP Wu LM Wei JQ. Effects of Chinese propolis in protecting bovine mammary epithelial cells against mastitis pathogens-induced cell damage. Mediators inflammation. (2016) 2016:8028291. doi: 10.1155/2016/8028291 PMC4940570 27433029

[67] Jin X Wang K Liu H Hu F Zhao F Liu J. Protection of bovine mammary epithelial cells from hydrogen peroxide-induced oxidative cell damage by resveratrol. Oxid Med Cell Longevity. (2016) 2016:2572175. doi: 10.1155/2016/2572175 PMC4707352 26962394

[68] Gao XJ Guo MY Zhang ZC Wang TC Cao YG Zhang NS. Bergenin plays an anti-inflammatory role via the modulation of MAPK and NF-κB signaling pathways in a mouse model of LPS-induced mastitis. Inflammation. (2015) 38:1142–50. doi: 10.1007/s10753-014-0079-8 25487780

[69] Ma X Xu S Li J Cui L Dong J Meng X. Selenomethionine protected BMECs from inflammatory injury and oxidative damage induced by Klebsiella pneumoniae by inhibiting the NF-κB and activating the Nrf2 signaling pathway. Int Immunopharmacology. (2022) 109027. doi: 10.1016/j.intimp.2022.109027 35820365

[70] Ma Y Ma X An Y Sun Y Dou W Li M. Green tea polyphenols alleviate hydrogen peroxide-induced oxidative stress, inflammation, and apoptosis in bovine mammary epithelial cells by activating erk1/2–nfe2l2–hmox1 pathways. Front Veterinary Science. (2022) 8:804241. doi: 10.3389/fvets.2021.804241 PMC8821889 35146014

[71] Zhu G Sui S Shi F Wang Q. Inhibition of USP14 suppresses ferroptosis and inflammation in LPS-induced goat mammary epithelial cells through ubiquitylating the IL-6 protein. Hereditas. (2022) 159:21. doi: 10.1186/s41065-022-00235-y 35549778 PMC9102600

[72] Dai H Coleman DN Hu L Martinez-Cortés I Wang M Parys C. Methionine and arginine supplementation alter inflammatory and oxidative stress responses during lipopolysaccharide challenge in bovine mammary epithelial cells *in vitro* . J Dairy Sci. (2020) 103:676–89. doi: 10.3168/jds.2019-16631 31733877

[73] Fusco R Cordaro M Siracusa R Peritore AF D’Amico R Licata P. Effects of hydroxytyrosol against lipopolysaccharide-induced inflammation and oxidative stress in bovine mammary epithelial cells: A natural therapeutic tool for bovine mastitis. Antioxidants. (2020) 9:693. doi: 10.3390/antiox9080693 32756342 PMC7464001

[74] Guo W Liu J Sun J Gong Q Ma H Kan X. Butyrate alleviates oxidative stress by regulating NRF2 nuclear accumulation and H3K9/14 acetylation via GPR109A in bovine mammary epithelial cells and mammary glands. Free Radical Biol Med. (2020) 152:728–42. doi: 10.1016/j.freeradbiomed.2020.01.016 31972340

[75] Shi H Guo X Yan S Guo Y Shi B Zhao Y. VA inhibits LPS-induced oxidative stress via modulating Nrf2/NF-κB-signalling pathways in bovine mammary epithelial cells. Ital J Anim Science. (2019) 18:1099–110. doi: 10.1080/1828051X.2019.1619490

[76] Shi HY Yan SM Guo YM Zhang BQ Guo XY Shi BL. Vitamin A pretreatment protects NO-induced bovine mammary epithelial cells from oxidative stress by modulating Nrf2 and NF-κB signaling pathways. J Anim Science. (2018) 96:1305–16. doi: 10.1093/jas/sky037 PMC6140872 29669072

[77] Wang F Zhao Y Chen S Chen L Sun L Cao M. Astragaloside IV alleviates ammonia-induced apoptosis and oxidative stress in bovine mammary epithelial cells. Int J Mol Sci. (2019) 20:600. doi: 10.3390/ijms20030600 30704086 PMC6386910

[78] Yu GM Tan W. Melatonin inhibits lipopolysaccharide-induced inflammation and oxidative stress in cultured mouse mammary tissue. Mediators Inflammation. (2019) 2019(1):8597159. doi: 10.1155/2019/8597159 PMC6390262 30890898

[79] Lyu C Yuan B Meng Y Cong S Che H Ji X. Puerarin alleviates H2O2-induced oxidative stress and blood–milk barrier impairment in dairy cows. Int J Mol Sci. (2023) 24:7742. doi: 10.3390/ijms24097742 37175449 PMC10178507

[80] Ali I Li C Kuang M Shah AU Shafiq M Ahmad MA. Nrf2 Activation and NF-Kb & caspase/bax signaling inhibition by sodium butyrate alleviates LPS-induced cell injury in bovine mammary epithelial cells. Mol Immunol. (2022) 148:54–67. doi: 10.1016/j.molimm.2022.05.121 35671559

[81] Meng M Huo R Wang Y Ma N Shi X Shen X. Lentinan inhibits oxidative stress and alleviates LPS-induced inflammation and apoptosis of BMECs by activating the Nrf2 signaling pathway. Int J Biol Macromolecules. (2022) 222:2375–91. doi: 10.1016/j.ijbiomac.2022.10.024 36243161

[82] Wang N Zhu Y Li D Basang W Huang Y Liu K. 2-methyl nonyl ketone from houttuynia cordata thunb alleviates LPS-induced inflammatory response and oxidative stress in bovine mammary epithelial cells. Front Chem. (2022) 9:793475. doi: 10.3389/fchem.2021.793475 35174140 PMC8842123

[83] Zhao N Yang Y Xu H Li L Hu Y Liu E. Betaine protects bovine mammary epithelial cells against LPS-induced inflammatory response and oxidative damage via modulating NF-κB and Nrf2 signalling pathway. Ital J Anim science. (2022) 21:859–69. doi: 10.1080/1828051X.2022.2070035

[84] Guo W Li W Su Y Liu S Kan X Ran X. GPR109A alleviate mastitis and enhances the blood milk barrier by activating AMPK/Nrf2 and autophagy. Int J Biol Sci. (2021) 17:4271. doi: 10.7150/ijbs.62380 34803497 PMC8579459

[85] Li R Fang H Shen J Jin Y Zhao Y Wang R. Curcumin alleviates LPS-induced oxidative stress, inflammation and apoptosis in bovine mammary epithelial cells via the NFE2L2 signaling pathway. Toxins. (2021) 13:208. doi: 10.3390/toxins13030208 33809242 PMC7999830

[86] Liu M Zhang C Xu X Zhao X Han Z Liu D. Ferulic acid inhibits LPS-induced apoptosis in bovine mammary epithelial cells by regulating the NF-κB and Nrf2 signalling pathways to restore mitochondrial dynamics and ROS generation. Veterinary Res. (2021) 52:1–1. doi: 10.1186/s13567-021-00973-3 PMC8278735 34256834

[87] Liu S Guo W Jia Y Ye B Liu S Fu S. Menthol targeting AMPK alleviates the inflammatory response of bovine mammary epithelial cells and restores the synthesis of milk fat and milk protein. Front Immunol. (2021) 12:782989. doi: 10.3389/fimmu.2021.782989 35003099 PMC8727745

[88] Sun Y Wu Y Wang Z Chen J Yang Y Dong G. Dandelion extract alleviated lipopolysaccharide-induced oxidative stress through the Nrf2 pathway in bovine mammary epithelial cells. Toxins. (2020) 12:496. doi: 10.3390/toxins12080496 32752301 PMC7472369

[89] Chen P Yang J Wu N Han B Kastelic JP Gao J. Streptococcus lutetiensis induces autophagy via oxidative stress in bovine mammary epithelial cells. Oxid Med Cell Longevity. (2022) 72022:2549772. doi: 10.1155/2022/2549772 PMC8843784 35178153

[90] Cheng X Aabdin ZU Wang Y Ma N Dai H Shi X. Glutamine pretreatment protects bovine mammary epithelial cells from inflammation and oxidative stress induced by γ-d-glutamyl-meso-diaminopimelic acid (iE-DAP). J Dairy Science. (2021) 104:2123–39. doi: 10.3168/jds.2020-18402 33358155

[91] Li Y Han N Hou P Zhao FQ Liu H. Roles of MAPK and Nrf2 signaling pathways in quercetin alleviating redox imbalance induced by hydrogen peroxide in mammary epithelial cells. Anim Nutriomics. (2024) 1:e1. doi: 10.1017/anr.2024.2

[92] Xu M Che L Gao K Wang L Yang X Wen X. Taurine alleviates oxidative stress in porcine mammary epithelial cells by stimulating the Nrf2-MAPK signaling pathway. Food Sci Nutr. (2023) 11:1736–46. doi: 10.1002/fsn3.v11.4 PMC10084955 37051345

[93] Kan X Liu J Chen Y Guo W Xu D Cheng J. Myricetin protects against H2O2-induced oxidative damage and apoptosis in bovine mammary epithelial cells. J Cell Physiol. (2021) 236:2684–95. doi: 10.1002/jcp.v236.4 32885418

[94] Perruchot MH Gondret F Robert F Dupuis E Quesnel H Dessauge F. Effect of the flavonoid baicalin on the proliferative capacity of bovine mammary cells and their ability to regulate oxidative stress. PeerJ. (2019) 7:e6565. doi: 10.7717/peerj.6565 30863682 PMC6407502

[95] Sun X Jia H Xu Q Zhao C Xu C. Lycopene alleviates H2O2-induced oxidative stress, inflammation and apoptosis in bovine mammary epithelial cells via the NFE2L2 signaling pathway. Food Funct. (2019) 10:6276–85. doi: 10.1039/C9FO01922G 31576860

[96] Shao D Gao Z Zhao Y Fan M Zhao X Wei Q. Sulforaphane suppresses H2O2-induced oxidative stress and apoptosis via the activation of AMPK/NFE2L2 signaling pathway in goat mammary epithelial cells. Int J Mol Sci. (2023) 24:1070. doi: 10.3390/ijms24021070 36674585 PMC9867344

[97] Zhang J Wang J Fang H Yu H Zhao Y Shen J. Pterostilbene inhibits deoxynivalenol-induced oxidative stress and inflammatory response in bovine mammary epithelial cells. Toxicon. (2021) 189:10–8. doi: 10.1016/j.toxicon.2020.11.002 33181164

[98] Xu J Wang XL Zeng HF Han ZY. Methionine alleviates heat stress-induced ferroptosis in bovine mammary epithelial cells through the Nrf2 pathway. Ecotoxicology Environ Safety. (2023) 256:114889. doi: 10.1016/j.ecoenv.2023.114889 37079940

[99] Wang H Hao W Yang L Li T Zhao C Yan P. Procyanidin B2 alleviates heat-induced oxidative stress through the Nrf2 pathway in bovine mammary epithelial cells. Int J Mol Sci. (2022) 23:7769. doi: 10.3390/ijms23147769 35887117 PMC9316217

[100] Wang Y Wang HL Xing GD Qian Y Zhong JF Chen KL. S-allyl cysteine ameliorates heat stress-induced oxidative stress by activating Nrf2/HO-1 signaling pathway in BMECs. Toxicol Appl Pharmacol. (2021) 416:115469. doi: 10.1016/j.taap.2021.115469 33640343

[101] Li C Wang Y Li L Han Z Mao S Wang G. Betaine protects against heat exposure–induced oxidative stress and apoptosis in bovine mammary epithelial cells via regulation of ROS production. Cell Stress Chaperones. (2019) 24:453–60. doi: 10.1007/s12192-019-00982-4 PMC6439124 30805833

[102] Ding H Li Y Zhao C Yang Y Xiong C Zhang D. Rutin supplementation reduces oxidative stress, inflammation and apoptosis of mammary gland in sheep during the transition period. Front veterinary science. (2022) 279:907299. doi: 10.3389/fvets.2022.907299 PMC9196631 35711805

[103] Lebda MA Elmassry IH Taha NM Elfeky MS. Nanocurcumin alleviates inflammation and oxidative stress in LPS-induced mastitis via activation of Nrf2 and suppressing TLR4-mediated NF-κB and HMGB1 signaling pathways in rats. Environ Sci pollut Res. (2022) F1:1–2. doi: 10.1007/s11356-021-16309-9 34482462

[104] Gross JJ Grossen-Rösti L Héritier R Tröscher A Bruckmaier RM. Inflammatory and metabolic responses to an intramammary lipopolysaccharide challenge in early lactating cows supplemented with conjugated linoleic acid. J Anim Physiol Anim Nutr. (2018) 102:102–48. doi: 10.1111/jpn.2018.102.issue-2 29178459

[105] Hanschke N Kankofer M Ruda L Höltershinken M Meyer U Frank J. The effect of conjugated linoleic acid supplements on oxidative and antioxidative status of dairy cows. J Dairy Sci. (2016) 99:8090–102. doi: 10.3168/jds.2015-10685 27497903

[106] Basiricò L Morera P Dipasquale D Tröscher A Serra A Mele M. Conjugated linoleic acid isomers strongly improve the redox status of bovine mammary epithelial cells (BME-UV1). J Dairy Sci. (2015) 98:7071–82. doi: 10.3168/jds.2015-9787 26277317

[107] Ma N Wei G Zhang H Dai H Roy AC Shi X. Cis-9, trans-11 CLA alleviates lipopolysaccharide-induced depression of fatty acid synthesis by inhibiting oxidative stress and autophagy in bovine mammary epithelial cells. Antioxidants. (2021) 11:55. doi: 10.3390/antiox11010055 35052560 PMC8773093

[108] Shao D Shen W Miao Y Gao Z Pan M Wei Q. Sulforaphane prevents LPS-induced inflammation by regulating the Nrf2-mediated autophagy pathway in goat mammary epithelial cells and a mouse model of mastitis. J Anim Sci Biotechnol. (2023) 14:61. doi: 10.1186/s40104-023-00858-9 37131202 PMC10155371

[109] He X Wang J Sun L Ma W Li M Yu S. Wogonin attenuates inflammation and oxidative stress in lipopolysaccharide-induced mastitis by inhibiting Akt/NF-κB pathway and activating the Nrf2/HO-1 signaling. Cell Stress Chaperones. (2023) 28:989–99. doi: 10.1007/s12192-023-01391-4 PMC10746643 37910344

[110] Malik MU Hashmi N Khan M Aabdin ZU Sami R Aljahani AH. Nutraceutical effect of resveratrol on the mammary gland: focusing on the NF-κb/nrf2 signaling pathways. Animals. (2023) 13:1266. doi: 10.3390/ani13071266 37048522 PMC10093560

[111] Zheng Z Zheng Y Liang X Xue G Wu H. Sanguinarine enhances the integrity of the blood–milk barrier and inhibits oxidative stress in lipopolysaccharide-stimulated mastitis. Cells. (2022) 11:3658. doi: 10.3390/cells11223658 36429086 PMC9688596

[112] Zhao C Wu K Bao L Chen L Feng L Liu Z. Kynurenic acid protects against mastitis in mice by ameliorating inflammatory responses and enhancing blood-milk barrier integrity. Mol Immunol. (2021) 134–44. doi: 10.1016/j.molimm.2021.06.022 34247099

[113] Ran X Zhang Y Yang Y Hu G Liu J Hou S. Dioscin improves pyroptosis in LPS-induced mice mastitis by activating AMPK/Nrf2 and inhibiting the NF-κB signaling pathway. Oxid Med Cell longevity. (2020) 2020:1–25. doi: 10.1155/2020/8845521 PMC7790561 33488936

[114] Xu D Liu J Ma HE Guo W Wang J Kan X. Schisandrin A protects against lipopolysaccharide-induced mastitis through activating Nrf2 signaling pathway and inducing autophagy. Int Immunopharmacology. (2020) 78:105983. doi: 10.1016/j.intimp.2019.105983 31767544

[115] Zhao C Jiang P He Z Yuan X Guo J Li Y. Dimethyl itaconate protects against lippolysacchride-induced mastitis in mice by activating MAPKs and Nrf2 and inhibiting NF-κB signaling pathways. Microbial pathogenesis. (2019) 133:103541. doi: 10.1016/j.micpath.2019.05.024 31100405

[116] Li M Wang Z Fu S Sun N Li W Xu Y. Taurine reduction of injury from neutrophil infiltration ameliorates Streptococcus uberis-induced mastitis. Int Immunopharmacology. (2023) 124:111028. doi: 10.1016/j.intimp.2023.111028 37857121

[117] Zhao L Jin L Yang B. Diosmetin alleviates S. aureus-induced mastitis by inhibiting SIRT1/GPX4 mediated ferroptosis. Life Sci. (2023) 331:122060. doi: 10.1016/j.lfs.2023.122060 37652155

[118] Zhao L Jin L Yang B. Saikosaponin A alleviates Staphylococcus aureus-induced mastitis in mice by inhibiting ferroptosis via SIRT1/Nrf2 pathway. J Cell Mol Med. (2023) 27:3443–50. doi: 10.1111/jcmm.v27.22 PMC10660613 37644785

[119] Glorieux C Enríquez C González C Aguirre-Martínez G Buc Calderon P. The multifaceted roles of NRF2 in Cancer: friend or foe? Antioxidants. (2024) 13:70. doi: 10.3390/antiox13010070 38247494 PMC10812565

[120] Occhiuto CJ Moerland JA Leal AS Gallo KA Liby KT. The multi-faceted consequences of NRF2 activation throughout carcinogenesis. Molecules Cells. (2023) 46:176–86. doi: 10.14348/molcells.2023.2191 PMC10070161 36994476

[121] Wu S Lu H Bai Y. Nrf2 in cancers: A double-edged sword. Cancer Med. (2019) 8:2252–67. doi: 10.1002/cam4.2019.8.issue-5 PMC6536957 30929309

[122] Jeddi F Soozangar N Sadeghi MR Somi MH Samadi N. Contradictory roles of Nrf2/Keap1 signaling pathway in cancer prevention/promotion and chemoresistance. DNA repair. (2017) 54:13–21. doi: 10.1016/j.dnarep.2017.03.008 28415030

[123] Lin L Wu Q Lu F Lei J Zhou Y Liu Y. Nrf2 signaling pathway: current status and potential therapeutic targetable role in human cancers. Front Oncol. (2023) 13:1184079. doi: 10.3389/fonc.2023.1184079 37810967 PMC10559910

